# Time delays modulate the stability of complex ecosystems

**DOI:** 10.1038/s41559-023-02158-x

**Published:** 2023-08-17

**Authors:** Yuguang Yang, Kevin R. Foster, Katharine Z. Coyte, Aming Li

**Affiliations:** 1 Center for Systems and Control, College of Engineering, Peking University, Beijing, China; 2 Department of Biology, University of Oxford, Oxford, UK; 3 Department of Biochemistry, University of Oxford, Oxford, UK; 4 Division of Evolution and Genomic Sciences, Faculty of Biology, Medicine and Health, University of Manchester, Manchester, UK; 5 Center for Multi-Agent Research, Institute for Artificial Intelligence, Peking University, Beijing, China

**Keywords:** Theoretical ecology, Ecological modelling, Community ecology

## Abstract

What drives the stability, or instability, of complex ecosystems? This question sits at the heart of community ecology and has motivated a large body of theoretical work exploring how community properties shape ecosystem dynamics. However, the overwhelming majority of current theory assumes that species interactions are instantaneous, meaning that changes in the abundance of one species will lead to immediate changes in the abundances of its partners. In practice, time delays in how species respond to one another are widespread across ecological contexts, yet the impact of these delays on ecosystems remains unclear. Here we derive a new body of theory to comprehensively study the impact of time delays on ecological stability. We find that time delays are important for ecosystem stability. Large delays are typically destabilizing but, surprisingly, short delays can substantially increase community stability. Moreover, in stark contrast to delay-free systems, delays dictate that communities with more abundant species can be less stable than ones with less abundant species. Finally, we show that delays fundamentally shift how species interactions impact ecosystem stability, with communities of mixed interaction types becoming the most stable class of ecosystem. Our work demonstrates that time delays can be critical for the stability of complex ecosystems.

## Abstract (web summary)

Time delays in the responses of species to one another are expected to occur widely in nature. Using a new theoretical framework, the authors show that these delays can fundamentally shift how different communities respond to perturbations.

## Main

The biological world comprises complex communities containing many interacting species. A key property of these communities is their stability, which determines the ability of constituent species to recover following perturbations^1,2^. There has been an intense effort to understand what determines community stability^1–16^. In particular, a large body of theory has been developed, on the basis of early work by May^3^, to study the effect of factors including the strength and sign of species interactions^4–6,8,9,11,13^, spatial structure^12,15^ and community interaction structure^4,8,10,16^ on the stability of complex communities.

However, the overwhelming majority of work on community stability so far has assumed that the growth rate of any individual species within a community responds immediately to changes in the abundances of other community members^3–16^. In reality, these responses are expected to occur only after time delays. For example, consider one bacterium (for example, *Pseudomonas aeruginosa*) that makes a toxin that inhibits a second bacterial species (for example, *Staphylococcus aureus*)^17^. If *P. aeruginosa*’s abundance increases, the amount of toxin within the environment will also increase, but only after a delay imposed by the time for each new cell to begin toxin production. Moreover, there will probably be a further delay before this newly produced toxin affects members of *S. aureus*, due to the time for the toxin to enter *S. aureus*’s cells and exert its effect. As such, overall, there will probably be a considerable lag before increases in *P. aeruginosa* exert an effect on the dynamics of *S. aureus* (see Supplementary Note 7 for an illustrative mathematical model of this example). In general, across species, there are diverse reasons to expect delays, including age structure^2,18^, seasonality^2,18^ and various metabolic processes^19,20^.

Indeed, time delays have long been recognized as playing a central role in community dynamics^1,2,18–27^. Theoretical work on single-species and simple two- or three-species exploitative systems has suggested that time delays can have major effects on ecosystem stability^1,18,19,21–26^. Yet in contrast, work on large communities of randomly interacting species has suggested that delays in interspecies interactions do not impact stability qualitatively^27^. These differences suggest that the impacts of time delays probably depend upon the precise ecological properties of the community in question. However, we lack a comprehensive understanding of the impacts of time delays in ecology, particularly when it comes to complex communities.

Motivated by this gap, here we develop a systematic framework to comprehensively study the effects of time delays on the local asymptotic stability of any ecosystem. Mirroring classical work on exploitative systems^1,24^, our work suggests that large delays in the effects of species on one another are destabilizing. However, critically, we find that short delays have the opposite effect and can in fact stabilize ecosystems. We also find that while some rules of ecological stability, such as the effects of diversity, are robust to delays, other principles are changed. In particular, the introduction of time delays can alter which community type is the most stable, and dramatically alter the relationship between a species’ population size and stability. Our work demonstrates the importance of time delays for ecological stability and provides a general framework to understand their impacts.

## Results

### Time delays alter whether communities are stable

**Fig. 1:**
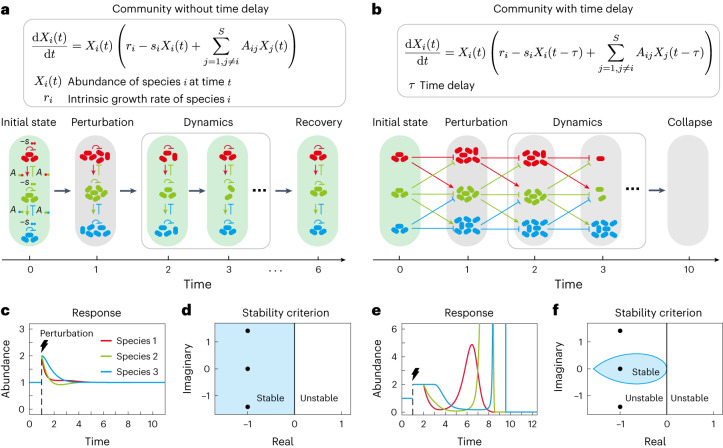
Illustration of the stability of communities with and without time delay. **a**, Schematic diagram of perturbing a three-species community without delay, whose dynamics follow the gLV model. Here, the change in a species’ abundance affects its own and other species’ abundances immediately. Sharp-head and blunt-head arrows represent positive and negative effects, respectively. **b**, Schematic diagram of perturbing the same three-species community when time delay is considered. In this case, species respond to changes in their own and other species’ abundances after a certain time delay (*τ* = 1). **c**, Response of a three-species delay-free community to external perturbations. Communities that recover to their previous state following perturbations are classified as stable and thus, this community is stable. **d**, Mathematically, a delay-free community is stable when all eigenvalues of the community matrix (given in equation (2)) have negative real parts, namely, all eigenvalues locate in the left half of the complex plane. For the three-species community shown in **c**, eigenvalues are depicted by black dots. **e**, The same three-species community as shown in **c** and **d** is unstable in the face of perturbations once time delay is introduced, as its eigenvalues lie outside the stability region (blue region in **f**). Here we set *r*_1_ = 2, *r*_2_ = 1, *r*_3_ = 0, *s*_*i*_ = 1, *A*_12_ = *A*_23_ = −1, *A*_21_ = *A*_32_ = 1, *A*_13_ = *A*_31_ = 0 for the three-species system. In numerical simulations, we regard a species as extinct if its abundance is <0.01.

Following the canonical framework of May and others^1–16,19,22,28,29^, we model an ecological community composed of *S* interacting species as a continuous-time autonomous dynamical system (Fig. 1),

1 $$\frac{\,{{\mbox{d}}}\,{{{\boldsymbol{X}}}}\left(t\right)}{\,{{\mbox{d}}}t}={{\mbox{diag}}}\,\left({{{\boldsymbol{X}}}}\left(t\right)\right){{{\boldsymbol{f}}}}\left({{{\boldsymbol{X}}}}\left(t-\tau \right)\right),$$

where ${{{\boldsymbol{X}}}}(t)={\left({X}_{1}\left(t\right),{X}_{2}\left(t\right),\cdots ,{X}_{S}\left(t\right)\right)}^{\text{T}}\in {{\mathbb{R}}}^{S}$ is an *S*-dimensional vector whose element ${X}_{i}\left(t\right)$ represents the abundance of species *i* at time *t*, ${{\mbox{diag}}}\,\left({{{\boldsymbol{X}}}}\left(t\right)\right)$ is a square diagonal matrix with the entries of ${{{\boldsymbol{X}}}}\left(t\right)$ on the main diagonal and all other entries outside the main diagonal set to zero, and ${{{\boldsymbol{f}}}}\left({{{\boldsymbol{X}}}}\left(t-\tau \right)\right)$ encodes the underlying ecological network and interactions. When time delays are absent (that is, *τ* = 0), equation (1) becomes the canonical delay-free case, where species respond immediately to abundance changes (Fig. 1a). Importantly, in this framework, whenever the *τ* is non-zero, species will no longer respond instantaneously to changes in their own or others’ abundances and instead respond only after a delay (Fig. 1b). If ${{{{\boldsymbol{X}}}}}^{\,{* }}$ > **0** satisfies ${{{\boldsymbol{f}}}}\left({{{{\boldsymbol{X}}}}}^{\,{* }}\right)={{{\bf{0}}}}$, it is defined as a feasible coexistence equilibrium^29^, where each species has a positive abundance.

Within theoretical ecology, multiple measures of the stability of an ecosystem exist^1,2,28,30–32^, but a key measure is the ability of an ecological system to return to an equilibrium in the face of external perturbations, also termed local asymptotic stability^1–16,18–27^. In unstable systems, infinitesimal perturbations can drive the system away from its feasible coexistence equilibrium and possibly lead to loss of species. Following classic stability analysis methods, whether a feasible coexistence equilibrium ${{{{\boldsymbol{X}}}}}^{\,{* }}$ is stable can be determined by the behaviour around this equilibrium. This behaviour is captured by linearizing equation (1) around the corresponding equilibrium (see Methods and Supplementary Note 1) as

2 $$\frac{\,{{\mbox{d}}}\,{{{\boldsymbol{x}}}}(t)}{\,{{\mbox{d}}}\,t}={{{\boldsymbol{M}}}}{{{\boldsymbol{x}}}}(t-\tau ),$$

where ${{{\boldsymbol{x}}}}\left(t\right)={{{\boldsymbol{X}}}}\left(t\right)-{{{{\boldsymbol{X}}}}}^{\,{* }}$ captures deviation from equilibrium abundance and ***M*** is the so-called ‘community matrix’ whose element *M*_*ij*_ represents the effect that species *j* has on species *i* around the equilibrium^1–15^.

For delay-free systems, stability can be determined by examining the maximum real part of the eigenvalues of ***M*** (that is, $\,{{\mbox{Re}}}\,\left({\lambda }_{1}\right)$). Specifically, provided $\,{{\mbox{Re}}}\,\left({\lambda }_{1}\right) < 0$, the system will be stable (Fig. 1c,d)^1–16,28,32^. However, the introduction of time delays (*τ* > 0) dramatically changes which communities are stable, often rendering previously stable communities unstable (Fig. 1e). In other words, the stability of ecological systems with time delays cannot be determined by the canonical method of identifying the maximum real eigenvalue^19,22,27,33^.

While the maximum real eigenvalue is no longer predictive of stability, in Methods we show that communities with time delays will be stable provided that all roots of the characteristic equation

3 $$H(z)=z-\lambda {{{\mbox{e}}}}^{-z\tau }=0,$$

have negative real parts. This requirement reduces the size of the region where stability is predicted within the complex plane, such that to ensure stability all eigenvalues *λ* of ***M*** now need to be located in a teardrop-shaped region defined by Re(*z*) < 0, satisfying equation (3) (blue region in Fig. 1f, see Methods). As illustrated in a three-species toy example in Fig. 1f, if any eigenvalues lie outside of the teardrop-shaped region, the system will be unstable and unable to recover following a perturbation, even if all eigenvalues lie within the left half complex plane (Fig. 1e). Note that when time delays are absent (*τ* = 0), this teardrop-shaped region expands to the whole left half complex plane (Fig. 1d) and thus, the stability criterion degenerates to the delay-free case.

### Metric of ecological stability with time delays

While the teardrop-shaped domain enables us to make the binary classification of whether or not a given community is stable, it does not allow us to compare the relative stability of different communities. That is, it cannot tell us whether one community is more stable than another. In the context of ecology, the degree of stability is commonly evaluated by recovery time: the time for a perturbation to decay to a specified fraction of its initial size^30,31^. Systems that need less recovery time are classified as more stable than systems that need more recovery time.

**Fig. 2:**
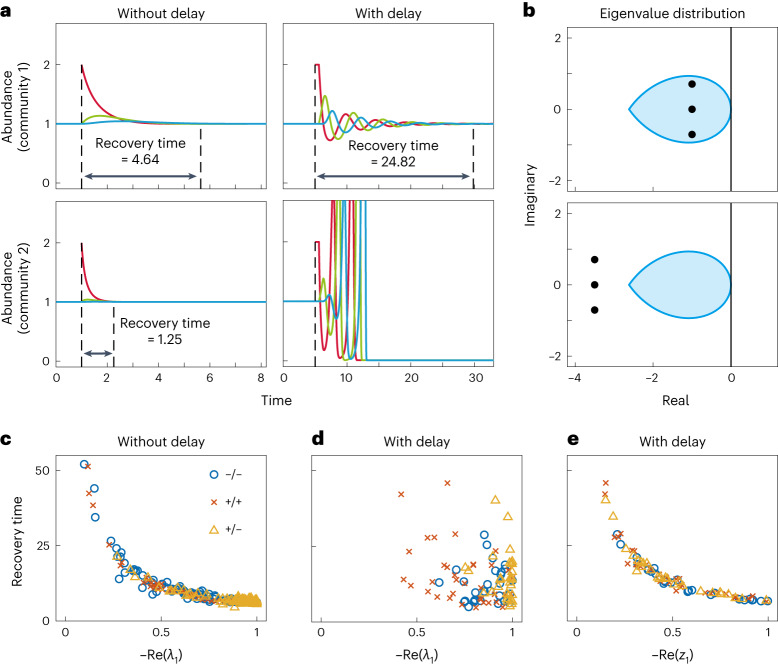
Quantifying the stability of time-delayed ecosystems. **a**, The responses of two three-species communities to perturbations before (left column) and after (right column) we introduce time delay. When time delay is absent, community 2 is more stable since the recovery time is shorter. When time delay exists, community 1 is stable while community 2 is unstable. Here we deem that the community recovers to the equilibrium if the deviation of each species’ abundance from its equilibrium is <0.1% and give the recovery time accordingly. **b**, Black dots represent the eigenvalues of these two communities. The blue teardrop-shaped area represents the stability region when time delay exists. The eigenvalues of community 1 locate in this region, indicating that it is stable in both cases. The eigenvalues of community 2 locate outside of this region, indicating that it is stable without delay but unstable when delay exists. **c**, The inverse proportional relationship between recovery time and the maximum real part of eigenvalues (that is, $-\,{{\mbox{Re}}}\,\left({\lambda }_{1}\right)$) of ***M*** (given in equation (2)) when time delay is absent. This was obtained by perturbing different types (competitive −/−, mutualistic +/+ and exploitative +/−) of three-species communities, suggesting that $-\,{{\mbox{Re}}}\,\left({\lambda }_{1}\right)$ quantifies the stability of delay-free systems. **d**, When we impose random time delays chosen from $\left[0.5,1\right]$ to communities in **c**, the inverse proportional relationship disappears, suggesting that $-\,{{\mbox{Re}}}\,\left({\lambda }_{1}\right)$ cannot quantify the stability of time-delayed systems. **e**, The inverse proportional relationship between recovery time of time-delayed systems and the maximum real part of the characteristic roots of equation (3) (that is, $-\,{{\mbox{Re}}}\,\left({z}_{1}\right)$), indicating that the stability of time-delayed systems is quantified by $-\,{{\mbox{Re}}}\,\left({z}_{1}\right)$. Simulations are based on the gLV model shown in Fig. 1. In **a** and **b**, we set *r*_1_ = 1.5, *r*_2_ = 1, *r*_3_ = 0.5, *s*_*i*_ = 1 for community 1; *r*_1_ = 4, *r*_2_ = 3.5, *r*_3_ = 3, *s*_*i*_ = 3.5 for community 2; and *A*_12_ = *A*_23_ = −0.5, *A*_21_ = *A*_32_ = 0.5, *A*_13_ = *A*_31_ = 0 for both communities. The time delay considered in **a** and **b** is 1. In **c**–**e**, we consider different types of fully connected three-species community with ${s}_{i}=1,\left\vert {A}_{ij,i\ne j}\right\vert$ is randomly chosen from [0,1] and equilibrium abundance of each species is 1.

In delay-free systems, this recovery time can be quantified by the maximum real part of the eigenvalues of the community matrix ***M*** (that is, $\,{{\mbox{Re}}}\,\left({\lambda }_{1}\right)$)^34^. $-\,{{\mbox{Re}}}\,\left({\lambda }_{1}\right)$ depicts the asymptotic rate at which perturbations to a stable delay-free ecosystem decay (Methods); thus, the more negative $\,{{\mbox{Re}}}\,\left({\lambda }_{1}\right)$ is, the shorter the recovery time, hence the more stable the system is (left column in Fig. 2a,b). However, examining the dynamics of a simple three-species system demonstrates that this is no longer the case for time-delayed systems (right column in Fig. 2a). Here, the introduction of time delays renders previously highly stable communities ($\,{{\mbox{Re}}}\,\left({\lambda }_{1}\right) < < 0$) far less stable than communities with less negative $\,{{\mbox{Re}}}\,\left({\lambda }_{1}\right)$ (Fig. 2b). Crucially, systematically varying community parameters demonstrates that this is a general phenomenon—introducing time delays breaks down the relationship between the magnitude of $\,{{\mbox{Re}}}\,\left({\lambda }_{1}\right)$ and the corresponding recovery time (Fig. 2d).

While the introduction of time delays disrupts the relationship between $-\,{{\mbox{Re}}}\,\left({\lambda }_{1}\right)$ and recovery time, we find that this role is instead taken by $-\,{{\mbox{Re}}}\,\left({z}_{1}\right)$ (*z*_1_ is the characteristic root with the maximal real part among all roots of equation (3), see Methods). That is, as shown by numerical simulations, the recovery time of time-delayed systems and $-\,{{\mbox{Re}}}\,\left({z}_{1}\right)$ shows the same inverse proportional relationship (Fig. 2e) as that between the recovery time of delay-free systems and $-\,{{\mbox{Re}}}\,\left({\lambda }_{1}\right)$ (Fig. 2c). Namely, a more negative $\,{{\mbox{Re}}}\,\left({z}_{1}\right)$ corresponds to a shorter recovery time. It is worth noting that a positive $\,{{\mbox{Re}}}\,\left({z}_{1}\right)$ (that is, local instability) does not necessarily guarantee subsequent system collapse and as with delay-free systems^35^, we can observe ‘unstable coexistence’ conditions wherein species abundances fluctuate either uniformly or chaotically about the equilibrium (Supplementary Fig. 22). However, anecdotally, we find that the likelihood of observing such communities decreases with increasing $\,{{\mbox{Re}}}\,\left({z}_{1}\right)$ (Supplementary Fig. 22). Such unstable coexistence can refer to stable limit cycles due to the introduction of time delays, although testing for their presence might not be analytically tractable. Moreover, time delays make chaos easier to generate^19^, where species can also persist^35^.

**Fig. 3:**
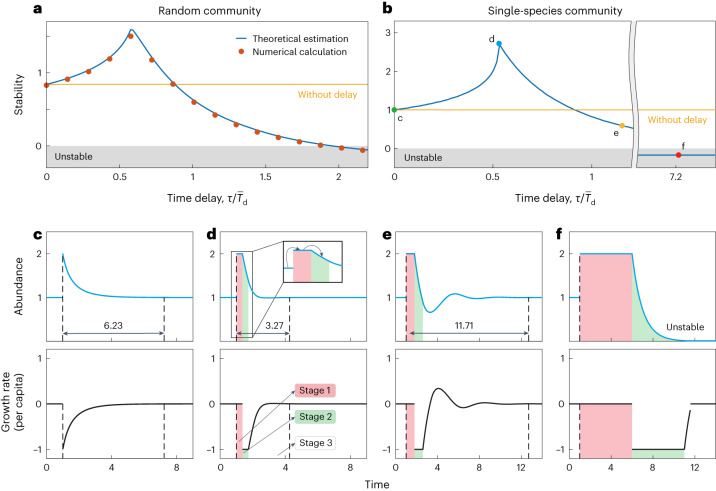
Moderate time delay stabilizes the ecosystem. **a**, The level of stability for random communities changes with the relative length of time delay, which is defined by the length of time delay *τ* divided by the average doubling time ${\overline{T}}_{\rm{d}}$ (given in Methods) of a random gLV system (see Fig. 1). The orange line indicates the stability level when time delay is absent. Red dots are results from numerical calculations where each dot is obtained from an average of 50 communities with interaction parameters drawn from the same distributions, and the blue line is from our theoretical estimation (given in Methods). **b**, The non-monotonic relation between stability and time delay also occurs in a single-species community. For different levels of time delay (dots c to f), we plot the response of the community after perturbation in **c**–**f** accordingly. **c**–**f**, Top: abundance dynamics. Bottom: the corresponding growth rate per capita. The response process can be divided into three stages. In the first stage (red region), the growth rate per capita remains 0. In the second stage (green region), the growth rate per capita is a negative constant. In the third stage (white region between dashed lines), the growth rate per capita changes over time. The subplot in **d** illustrates the delayed response of species abundance, namely, each current state is affected by the preceding state at *τ* time. Note that when the species is extinct, we set the growth rate per capita to 0. In **a**, community parameters are *S* = 100, *C* = 0.1, *s* = 1, *σ* = 0.05. In **b**, we set the per capita self-regulation strength of this single-species population as *s* = 1 and numerical simulations in **c**–**f** were performed with the unit intrinsic growth rate and carrying capacity.

Having established that $-\,{{\mbox{Re}}}\,\left({z}_{1}\right)$ quantifies the stability of time-delayed systems, we then face the challenge of determining $\,{{\mbox{Re}}}\,\left({z}_{1}\right)$ for large complex ecosystems. This requires solving equation (3) *S* times (since ***M*** has *S* eigenvalues), making stability analysis of time-delayed complex ecosystems a non-deterministic polynomial hard (NP-hard) problem^36^ that cannot be solved in polynomial time (that is, the time required to solve equation (3) is prohibitively long). To overcome this challenge, we developed a new, time-efficient way to estimate $\,{{\mbox{Re}}}\,\left({z}_{1}\right)$ from only the three endpoints of the eigenvalue distribution instead of all eigenvalues (see Methods and Supplementary Note 2). This method greatly reduces the computation time needed for stability analysis. Moreover, results from numerical simulations are in good agreement with the theoretical estimates (Fig. 3a and Supplementary Figs. 3, 6, 7 and 11).

### Short versus long delays have opposing impacts on stability

How do time delays impact ecological stability? Existing work^1,23,24^ on single-species communities has shown that long delays can lead to oscillatory overshoots in species abundances that decrease community stability, but whether this phenomenon is common to all delays or community types has until now remained unknown. To our surprise, our new framework reveals that the relationship between stability and time delay is non-monotonic. Specifically, as the magnitude of time delays increases, it first stabilizes and then destabilizes the original community (Fig. 3a). This non-monotonic relationship between delay length and stability holds regardless of interaction types, community connectance or community size (see Supplementary Figs. 6 and 7). Note that, in the figures, we quantify time delay relative to the average doubling time of a random generalized Lotka-Volterra (gLV) system^37^ (that is, ${\overline{T}}_{\rm{d}}$, see Methods and Supplementary Note 6) to put the delay on a biologically meaningful footing. For example, for bacteria that double every generation via binary fission, when $\tau /{\overline{T}}_{\rm{d}} < 1$, the delays in the system are less than one generation and so on.

While initially unexpected, the stabilizing effect of short delays has an intuitive basis. The relationship between the magnitude of the delay and stability is seen in single-species communities (Fig. 3b) as well as diverse communities. As such, we can use the single-species case to better understand the relationship (Fig. 3c–f). To do this, we focus here on a perturbation that increases population size, but similar logic can be derived for a downward perturbation. We divide the response process into three stages according to the per capita growth rate after perturbation, where the change in species abundance at time *t* is affected by the abundance at time *t* − *τ* due to the existence of the time delay. (1) Stage 1 (Fig. 3d–f, red region): here, the species’ per capita growth rate equals 0 for time *τ* because it is determined by the species’ abundance before the perturbation (subplot in Fig. 3d), during which the population rests at its equilibrium. During this stage, the species’ abundance remains at the perturbed level. (2) Stage 2 (Fig. 3d–f, green region): here, the species’ per capita growth rate is a negative constant for a length of time *τ*, because its growth rate is now determined by the perturbed abundance level (subplot in Fig. 3d) in stage 1. (3) Stage 3 (Fig. 3d–f, white region between dashed lines): here, the species’ per capita growth rate is determined by the species’ changing abundance in stages 2 and 3, and therefore itself keeps changing. This stage lasts until the species’ abundance has returned to equilibrium, or until the species has gone extinct, and is therefore typically the longest stage.

In a single-species situation without time delay, there is no stage 1 and no stage 2 (as *τ* = 0), thus the species recovers monotonically to equilibrium following the perturbation (Fig. 3c). For a small delay (Fig. 3d), the return to equilibrium is delayed by stage 1, but when the return begins (stage 2), the population is now always responding to a higher (past) abundance than it would without delays. As a result, the rate of return during this stage is much higher than in the no-delay case. By the time the system enters stage 3, it is almost at equilibrium; hence the overall effect is to reduce the recovery time (that is, increasing stability) compared with a delay-free system. With a longer time delay, however, we see a very different outcome (Fig. 3e). Here, the rapid return to equilibrium driven by stage 2 is maintained for a long period of time and can thus cause the species’ abundance to overshoot its initial equilibrium. In stage 3 the system begins to recover from this overshoot but does so again with a delay that is sufficient for it to again overshoot the equilibrium in the other direction, setting up a series of oscillations about the equilibrium. These behaviours mean that stage 3 is now much longer than without a time delay, and recovery time is therefore increased, resulting in a decrease in stability. Notably, this destabilizing oscillatory overshoot follows the same mechanism that has previously been recognized to drive instability in single-species systems^1,23,24^. Pushing the time delay further means the overshoot of the equilibrium in stage 2 can be so large that species go extinct (Fig. 3f), and the system is rendered unstable (see Supplementary Note 3 for a theoretical analysis of the single-species time-delayed system and see Supplementary Fig. 5 for detailed response processes under different time delays).

In short, the introduction of time delay prolongs the influence of a perturbation (that is, stage 2). However, when the time delay is small, this prolonged influence is in fact stabilizing because it pushes the species to return to equilibrium more quickly. As the time delay increases, the outcome shifts and one sees the more expected oscillation in abundances because of a mismatch between instant abundance and growth rate. When the time delay is large enough, this prolonged influence leads to community collapse.

Our main analysis involves several simplifying assumptions, most notably that (1) members of a community each experience the same level of time delays in their population and (2) regardless of the true nature of the interactions within a community, an equilibrium dynamics can be approximated by the simple, direct net effects of species on one another (for example, the *A*_*ij*_ terms of the gLV model). In reality, different species may experience different time delays and interactions between taxa can take highly complex functional forms, often mediated by external factors such as metabolites or toxins. Incorporating such inhomogeneous time delays is challenging, as it again returns us to an NP-hard problem^36^, while explicitly modelling more complex interactions may suggest that delays in species interactions are already implicitly embedded in the modelling framework. Nevertheless, we wanted to check the robustness of our results to such complexities. We therefore numerically studied the effects of both inhomogeneous delays and complex interaction forms using a simplified two-species case (see Supplementary Note 7). These analyses show that our predictions are robust to such changes in assumptions: in both cases where delays are on average short, they are stabilizing, but long delays drive instability (Supplementary Figs. 19 and 21, note that due to the increased complexity of inhomogeneous delays and modelling metabolite-mediated interactions, here we limit our analysis to just two species; it therefore remains an important open problem as to whether these results hold in more diverse communities).

### Delays change which type of community is the most stable

**Fig. 4:**
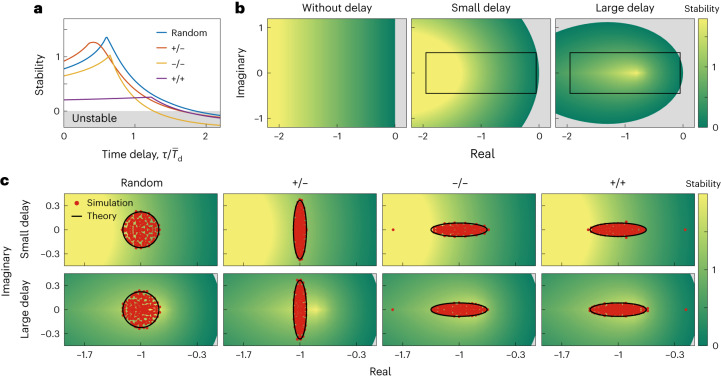
A diversity of interaction types can lead to a more stable ecological community. **a**, The relationship between stability and the intensity of time delay for different types of community (exploitative +/−, competitive −/−, mutualistic +/+ and a random mix of +/−, −/− and +/+). **b**, Stability contour plot in the complex plane at different intensities of time delay. Colours represent the level of stability. Darker green indicates lower stability; grey indicates instability. As the time delay increases, the curvature of the contour lines increases and the stability region shrinks. **c**, The eigenvalue distributions of different types of community with the stability contour plot for small (top) and large (bottom) time delay. The region discussed here is marked with the black rectangle in the middle and right panels of **b**. Red dots are the eigenvalues from numerical calculations, while black lines are boundaries predicted by theory (Methods). The parameters of all communities discussed here are the same as those in Fig. 3a except *S* = 200, and we set *τ* = 0.2 and 0.5 for small and large time delay, respectively.

A key question in ecology is how the interactions between individual species influence overall community stability^4–6,8,9,11,13^. In delay-free systems, the most stable communities are those in which all species interact in an exploitative manner (+/−)^4,9^, where a common intuition provided for this result is that +/− interactions drive direct negative feedbacks between species that help to promote stability. Our framework allows us to test whether this prediction holds for systems where there are time delays in the responses of species to one another. Importantly, we find that the introduction of time delays does indeed change this prediction. Specifically, we find that above a threshold in the delay length, the relative stability of exploitative communities drops sharply and becomes comparable to both competitive communities (−/−) and those containing a random mix of interaction types. Moreover, this leads to a large area of parameter space where random communities, that is, communities composed of a mix of +/+, −/− and +/− interactions, are the most stable systems (Fig. 4a).

What drives this complex relationship between interaction type, delay and stability? A key driver in the shift in the most stable community type is the rapid drop in the stability of exploitative communities with increasing delay. This drop has an intuitive basis because the introduction of time delay shifts exploitative interactions from being instantaneous negative feedbacks, which are highly stabilizing, to out-of-phase feedbacks, which are much less stabilizing. Introducing delays, therefore, has a stronger negative effect on the stability of exploitative interactions than the other interaction types, which helps to explain why the most stable community shifts to one with a random set of interaction types.

More formally, we can understand these patterns by looking at the shape of eigenvalue distributions for different systems and the stability contour plot (Fig. 4b,c, see Methods). Without delays, stability is determined by the rightmost eigenvalue of this distribution and, because the shape of the eigenvalue distribution changes with interaction type, so too does stability. For random communities, the eigenvalues are distributed in a circle with radius *R* (Fig. 4c, first column). For exploitative communities, the eigenvalues are distributed in a vertically stretched ellipse whose horizontal radius is smaller than *R* and whose vertical radius is larger than *R* (Fig. 4c, second column). For competitive communities and mutualistic communities (Fig. 4c, third and fourth columns), the eigenvalues can be divided into two parts: the bulk of the eigenvalues distributed in a horizontally stretched ellipse and an outlier (for competitive communities, this outlier is on the left side of the ellipse; for mutualistic communities, this outlier is on the right side). This horizontally stretched ellipse possesses a horizontal radius larger than *R* and a vertical radius smaller than *R*. Without delays, therefore, it is clear that the vertically stretched nature makes exploitative communities the most stable community.

The introduction of time delays distorts the stability contour plot (Fig. 4b). For small delays, these changes are relatively small (middle panel, Fig. 4b) and the stability of each type of community is still determined by the rightmost eigenvalue. As a result, exploitative communities are still the most stable (upper row, Fig. 4c). However, as the time delay increases, the stability contour plot changes further and the stability region shrinks (right panel, Fig. 4b). In the face of these changes, it is the eigenvalue distribution of the random communities that maps the best to high-stability regions of the contour plots (bottom row, Fig. 4c). As a result, it is random communities that show the highest stability for large time delays (see Supplementary Note 4 for details). Further analyses of communities with mixed interactions also show that communities with mixed interactions are more stable than communities with a single interaction type (Supplementary Fig. 11). In summary, we find that a diversity of interaction types can lead to a more stable community when there are time delays in the system.

### Time delays can destabilize large populations

**Fig. 5:**
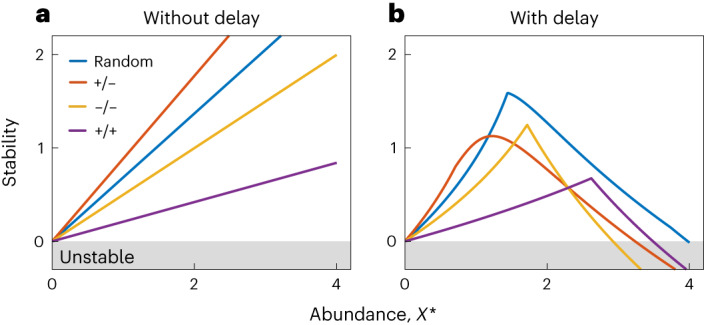
Time delays can destabilize large populations. The influence of species abundance on stability. **a**, Without delays, stability increases linearly as species abundance increases. **b**, With delays, this relationship becomes non-monotonic and large population sizes can destabilize the community. The parameters are the same as those in Fig. 3a, all species share the same equilibrium abundance ${X}^{\,{* }}$ and we set *τ* = 0.3 in **b**.

Identifying populations that are at risk of extinction is a central goal of ecology^38–41^. A key intuitive result from existing theory is that species or communities with large population sizes at equilibrium are more stable^38–40^. Mathematically, this is because the eigenvalues of higher-abundance systems are typically situated more deeply in the left half of the complex plane, leading to a higher level of self-regulation and therefore a rapid recovery from perturbation (Fig. 5a). Using our framework, we can study the nature of this relationship for systems where there are time delays in species responses. In the analyses above, the community rests at the equilibrium point ${{{{\boldsymbol{X}}}}}^{\,{* }}$ = **1**. To study the impact of changing species abundances, therefore, we can systematically vary ${{{{\boldsymbol{X}}}}}^{\,{* }}$.

In contrast to delay-free systems, we find that time delays fundamentally change the relationship between average species abundances and stability (Fig. 5b). As species abundances increase, stability first increases and then decreases. Importantly, a consequence of this is that when species abundances are sufficiently large, the system loses stability entirely. This non-monotonic relationship also modulates the impact of different interaction types, with random communities outperforming exploitative systems in stability at high species abundances. Again, therefore, we find that a diversity of interaction types can make the most stable communities (see Supplementary Note 5 for details).

## Discussion

Time delays are expected for many ecological interactions and have long been considered important for ecosystem stability^1,2,18–27^. However, delays generate an NP-hard problem for diverse communities, which has limited our ability to study their impacts^36^. Here we overcome this problem with a novel estimation framework, which allows one to comprehensively analyse the stability of large complex systems with time delays. A key novel finding from our work is that the relationship between time delays and stability is non-monotonic, with small delays able to improve system stability. This result contrasts with the existing intuition from previous work that time delays are often destabilizing^1,18,19,21–26^. We believe that the reason for this discrepancy is primarily due to the focus in past work on large time delays, where we also find that delays can be destabilizing.

Our work also reveals that with sufficient delay, the identity of the most stable communities changes to that of communities with a diversity of interaction types. Moreover, we find that large population sizes have the potential to be problematic for stability. This outcome occurs because large population sizes can generate strong negative feedbacks that, in the presence of delays, can cause large population fluctuations. This result is important, as it brings into question the commonly held intuition that large populations are the least threatened by extinctions.

Building on a large body of previous work^1–16,18–27^, here we have focused on local asymptotic stability as a measure of ecological stability. This measure assesses solely whether communities will be able to return to their initial equilibria following small perturbations (and how this ability is altered by the presence of time delays). However, there are many other measures of ecosystem stability^2,28,30–32^, including resistance^2,30,31^ (how much are abundances changed by a given disturbance?), structural stability^28^ (how broad are the conditions for the existence of feasible equilibria?), persistence^2,30,31^ (will any species go extinct following a perturbation?) and more. While these measures often correlate with one another^2,9,30,31^, there is the potential for differences. For example, our observation of persistent yet unstable coexistence states illustrates how looking beyond local asymptotic stability can have value. Exploring whether and how these different stability measures are affected by time delays is therefore an interesting open question.

Our work has focused specifically on ‘delayed’ interactions, formally defined as situations where a change in the abundance of one species is only felt by another after a certain time lag. However, there are other ways that the historic state of an ecosystem can influence future dynamics. One such example is ‘ecological memory’ wherein changes to an ecosystem can have prolonged impacts on future dynamics, long after the initial change occurs^42^. For example, this could occur due to species progressively altering the abiotic properties of the environments in which they reside. As with long time delays, ecological memory has been found to destabilize communities, increasing the time taken to recover from perturbation (although memory can also increase community resistance). However, current analyses^42^ of memory effects are restricted to small, purely competitive ecosystems, thus exploring how this and other forms of hysteresis impact the stability of different community types, or combine with classic interaction delays, presents an exciting open question for the future.

In conclusion, we find that time delays have the potential to rewrite the principles of ecological stability. Given that these delays are likely to be widespread, our work suggests that a better understanding of time delays and their impacts is an important goal for ecology.

## Methods

### Stability analysis of systems with time delay

For the system depicted in equation (1) resting at ${{{{\boldsymbol{X}}}}}^{\,{* }}$, the dynamics around ${{{{\boldsymbol{X}}}}}^{\,{* }}$ can be obtained by linearization^19,22^, which yields

$$\frac{\,{{\mbox{d}}}\,{{{\boldsymbol{x}}}}(t)}{\,{{\mbox{d}}}t}={{\mbox{diag}}}\,\left({{{{\boldsymbol{X}}}}}^{\,{* }}\right){\left.{{{\boldsymbol{J}}}}\right\vert }_{{{{{\boldsymbol{X}}}}}^{\,{* }}}{{{\boldsymbol{x}}}}(t-\tau ).$$

Here, ${{{\boldsymbol{x}}}}\left(t\right)={{{\boldsymbol{X}}}}\left(t\right)-{{{{\boldsymbol{X}}}}}^{\,{* }}$ describes the deviation from equilibrium abundance, and the entry ${J}_{ij}{\left.\right\vert }_{{{{{\boldsymbol{X}}}}}^{\,{* }}}={\left. \frac{ \partial {f}_{i}(t-\tau ) }{ \partial {X}_{j}\left(t-\tau \right)} \right\vert }_{{{{{\boldsymbol{X}}}}}^{\,{* }}}$ of ${\left.{{{\boldsymbol{J}}}}\right\vert }_{{{{{\boldsymbol{X}}}}}^{\,{* }}}$ captures the per capita interaction strength. We denote ${\left.{{{\boldsymbol{J}}}}\right\vert }_{{{{{\boldsymbol{X}}}}}^{\,{* }}}$ as ***J***, name ***J*** as the ‘interaction matrix’ and name the ‘community matrix’^3,4^${{{\boldsymbol{M}}}}=\,{{\mbox{diag}}}\,\left({{{{\boldsymbol{X}}}}}^{\,{* }}\right){{{\boldsymbol{J}}}}$, as we present in equation (2). For simplicity and in line with previous work^9,15^, initially we set ${{{{\boldsymbol{X}}}}}^{\,{* }}$ = **1**, meaning that all species have the same equilibrium abundance (later we explore the effect of population size by equally raising or lowering the abundance of all species in the community). It is interesting to note that previous theoretical results^14^ have shown that relaxing this assumption does not qualitatively impact the stability of delay-free systems, thus explicitly exploring the impact of similar variable abundances on the stability of time-delayed systems is an interesting open question.

The stability of the equilibrium can be determined by checking the roots of the following characteristic equation^19,22,27^:

$$\,{{\mbox{det}}}\,\left(z{{{\boldsymbol{I}}}}-{{{\boldsymbol{M}}}}{{{\mbox{e}}}}^{-z\tau }\right)=0.$$

Here ***I*** is an identity matrix and ***M*** is the community matrix defined above. When all roots *z* have negative real parts, the corresponding equilibrium ${{{{\boldsymbol{X}}}}}^{\,{* }}$ is stable. This equation further generates the decoupled characteristic equation (that is, equation (3), see Supplementary Note 1 for detailed derivation). When $\,{{\mbox{Re}}}\,\left(z\right)=0$, by solving the characteristic equation, we get the boundary

$$\tau =\frac{1}{\sqrt{{x}^{2}+{y}^{2}}}{{{\mbox{tan}}}}^{-1}\left(-\frac{x}{y}\right),$$

which encloses a teardrop-shaped region (Fig. 1f). If all eigenvalues *λ* of ***M*** fall inside this region when plotted as $\left(\,{{\mbox{Re}}}\,\left(\lambda \right),\,{{\mbox{Im}}}\,\left(\lambda \right)\right)$, all characteristic roots *z* will thus have negative parts, rendering a stable system. It is worth noting that when time delays are absent (*τ* = 0), equation (3) becomes $H\left(z\right)=z-\lambda =0$ and the stability criterion degenerates the delay-free case (that is, $\max \left(\,{{\mbox{Re}}}\,\left(\lambda \right)\right) < 0$).

### Constructing interaction matrices

Following the canonical framework in studying ecosystem stability^3,4,9^, we model different types of community by directly constructing the interaction matrix ***J***. For random communities, two species *i* and *j* interact with probability *C*, and the per capita interaction strengths *J*_*ij*_ and *J*_*ji*_ take the value of a random variable *Z* with mean 0 and variance *σ*^2^ respectively and independently. The diagonal terms *J*_*ii*_, representing self-regulation, are all set to −*s* (*s* > 0). For communities with complex interaction types, two species still interact with probability *C*. With probability *P*_m_, two species interact in a mutualistic manner, and the per capita interaction strengths *J*_*ij*_ and *J*_*ji*_ take the value of $$\left\vert Z\right\vert$$ respectively and independently. With probability *P*_c_, two species interact in a competitive manner, and the per capita interaction strengths *J*_*ij*_ and *J*_*ji*_ take the value of $$-\left\vert Z\right\vert$$ respectively and independently. With probability *P*_e_, two species interact in an exploitative manner, and the per capita interaction strengths *J*_*ij*_ and *J*_*ji*_ have opposite signs: one takes the value of $$\left\vert Z\right\vert$$ while the other takes the value of $$-\left\vert Z\,\right\vert$$. The diagonal terms *J*_*ii*_ are also set to −*s*. The statistical features of the interaction matrix can then be extracted for different types of community. For random communities, we have $${\mathbb{E}}\left(\,{J}_{ij,i\ne j}\right)=0,\,{{\mbox{Var}}}\,\left(\,{J}_{ij,i\ne j}\right)=$$$$C{\sigma }^{2},{\mathbb{E}}\left(\,{J}_{ij,i\ne j}\,{J}_{ji,i\ne j}\right)=0$$. For communities with complex interaction types, we have $${\mathbb{E}}\left(\,{J}_{ij,i\ne j}\right)=$$ $$C{\mathbb{E}}\left(\left\vert Z\right\vert \right)\left({P}_{{{{\rm{m}}}}}-{P}_{c}\right),\,{{\mbox{Var}}}\,\left(\,{J}_{ij,i\ne j}\right)$$$$=C{\sigma }^{2}-{\left({\mathbb{E}}\left(\,{J}_{ij,i\ne j}\right)\right)}^{2},{\mathbb{E}}\left(\,{J}_{ij,i\ne j}\,{J}_{ji,i\ne j}\right)=$$$$C{{\mathbb{E}}}^{2}\left(\left\vert Z\right\vert \right)\left({P}_{{{{\rm{m}}}}}+{P}_{{{{\rm{c}}}}}-{P}_{{{{\rm{e}}}}}\right)$$ .

### The metric of stability for time-delayed systems

Since the level of stability is normally evaluated by the recovery time^30,31^, here we offer the derivation of a new metric quantifying recovery time for time-delayed systems. The particular solution of equation (2) can be obtained through the combination of ${{{{\boldsymbol{\phi }}}}}_{i}(t)=\text{e}^{{z}_{i}t}{{{{\boldsymbol{k}}}}}_{i}\left(t\right)$ (*z*_*i*_ is the *i*th characteristic root of equation (3), ${{{{\boldsymbol{k}}}}}_{i}\left(t\right)$ is an *S*-dimensional vector whose elements are polynomials with respect to *t* and it is determined by the state before perturbation as well as the perturbation). That is, ${{{\boldsymbol{x}}}}\left(t\right)=\mathop{\sum }\nolimits_{i = 1}^{{n}_{r}}\text{e}^{{z}_{i}t}{{{{\boldsymbol{k}}}}}_{i}\left(t\right)$ (*n*_*r*_ is the number of characteristic roots).

When time delays are absent, equation (3) is not a quasi-polynomial and has *S* (number of species) characteristic roots (which are the eigenvalues of ***M***). Therefore, the solution of delay-free linearized systems can be represented as ${{{\boldsymbol{x}}}}\left(t\right)=\mathop{\sum }\nolimits_{i = 1}^{S}\text{e}^{{\lambda }_{i}t}{{{{\boldsymbol{k}}}}}_{i}\left(t\right)$. It is clear that the real parts of *λ* represent the decaying rates of each component of the solution. Among these eigenvalues, $-\,{{\mbox{Re}}}\,\left({\lambda }_{1}\right)$ (*λ*_1_ is the eigenvalue with the largest real part) is the lowest decaying rate and thus determines the final recovery time. Therefore, $-\,{{\mbox{Re}}}\,\left({\lambda }_{1}\right)$ is often used by ecologists to quantify the recovery time, and we have

$$\,{{\mbox{Stability}}}=-{{\mbox{Re}}}\,({\lambda }_{1}).$$

When time delays are considered, equation (3) has infinite number of roots^33^ and the particular solution takes the form ${{{\boldsymbol{x}}}}\left(t\right)=\mathop{\sum }\nolimits_{i = 1}^{\infty }\text{e}^{{z}_{i}t}{{{{\boldsymbol{k}}}}}_{i}\left(t\right)$. It is clear that the real parts of *z* represent the decaying rates, and $-\,{{\mbox{Re}}}\,\left({z}_{1}\right)$ now is the lowest decaying rate that quantifies stability. Hence, the stability of time-delayed systems can be defined as

$$\,{{\mbox{Stability}}}=-{{\mbox{Re}}}\,({z}_{1}).$$

### The contour plot of stability

To analyse the stability of large complex ecosystems with time delays, we depict the stability contour plot in the complex plane. Let $z=-\alpha +\text{i} \omega ,\alpha \in {{\mathbb{R}}}_{0}^{+},\omega \in {\mathbb{R}}$. Due to the symmetry of *z*, we only need to consider non-negative *ω*, namely for $\omega \in {{\mathbb{R}}}_{0}^{+}$. Substituting *z* = −*α* + i *ω* into equation (3), we arrive at the boundary for *α*-stability region

$$\left\{\begin{array}{l}\tau =\frac{1}{\omega }{{{\mbox{tan}}}}^{-1}\left(\frac{-\alpha y-\omega x}{-\alpha x+\omega y}\right),\\ \omega =\sqrt{{{{\mbox{e}}}}^{2\alpha \tau }({x}^{2}+{y}^{2})-{\alpha }^{2}}.\end{array}\right.$$

For *λ* inside this boundary, the corresponding $-\,{{\mbox{Re}}}\,\left({z}_{1}\right) > \alpha$. For *λ* on this boundary, the corresponding $-\,{{\mbox{Re}}}\,\left({z}_{1}\right)=\alpha$. For *λ* outside this boundary, the corresponding $-\,{{\mbox{Re}}}\,\left({z}_{1}\right) < \alpha$. By increasing *α* from 0 and plotting the corresponding *α*-stability boundary, the stability contour plot in the complex plane can be obtained and is presented in Fig. 4b. Indeed, these boundaries form contour lines in the contour plot of stability. As the intensity of time delay increases, the curvature of these contour lines increases together with the shrinkage of the stability region (Fig. 4b and Supplementary Fig. 8). We can see that eigenvalues closer to the boundary of the stability region lead to lower $-\,{{\mbox{Re}}}\,\left({z}_{1}\right)$, that is, lower stability level (see Supplementary Note 2 for more details).

### Estimating the stability of large complex ecosystems with time delays

Since the endpoints of the eigenvalue distribution are more likely to locate near the boundary of the stability region (see Supplementary Note 2 for more details), we only need to find three endpoints (leftmost, rightmost and uppermost) to estimate the stability of large complex ecosystems with time delays. For simplicity, here we denote some statistical features of the community matrix ***M*** by ${\mathbb{E}}\left({M}_{ij,i\ne j}\right)=E,\,{{\mbox{Var}}}\,\left({M}_{ij,i\ne j}\right)=V,{\mathbb{E}}\left({M}_{ij,i\ne j}{M}_{ji,i\ne j}\right)=\rho$.

For random communities^3,4,7,9^, eigenvalues of ***M*** are uniformly distributed in a circle with radius $\sqrt{SV}$ centred at (−*s*, 0) (see Supplementary Note 2 for detailed derivation). Therefore, the corresponding endpoints are

$$\left\{\begin{array}{l}{Q}_{{{{\rm{leftmost}}}}}\left(-s-\sqrt{SV},0\right),\\ {Q}_{{{{\rm{rightmost}}}}}\left(-s+\sqrt{SV},0\right),\\ {Q}_{{{{\rm{uppermost}}}}}\left(-s,\sqrt{SV}\right).\end{array}\right.$$

For communities with complex interaction types^4,7,9^, eigenvalues of ***M*** can be divided into two parts: the bulk of eigenvalues are distributed in an ellipse centred at (−*s* − *E*, 0) and an outlier. Lengths of the half-horizontal axis and the half-vertical axis of the ellipse are $\sqrt{SV}\left(1+\left(\rho -{E}^{2}\right)/V\right) {\rm{and}}\, {\sqrt{SV}}\left(1-\left({\rho} -{E}^{2}\right)/V\right)$, respectively. The outlier is ${Q}_{{{{\rm{outlier}}}}}\left(-s+\left(S-1\right)E,0\right)$ (see Supplementary Note 2 for detailed derivation). The corresponding endpoints are then

$$\left\{\begin{array}{l}{Q}_{{{{\rm{leftmost}}}}}\left(\min \left(-s-E-\sqrt{SV}\left(1+\frac{\rho -{E}^{2}}{V}\right),-s+(S-1)E\right),0\right),\\ {Q}_{{{{\rm{rightmost}}}}}\left(\max \left(-s-E+\sqrt{SV}\left(1+\frac{\rho -{E}^{2}}{V}\right),-s+(S-1)E\right),0\right),\\ {Q}_{{{{\rm{uppermost}}}}}\left(-s-E,\sqrt{SV}\left(1-\frac{\rho -{E}^{2}}{V}\right)\right).\end{array}\right.$$

Substituting these endpoints into the characteristic equation and calculating the corresponding *z*, we get the estimation for $-\max (\,{{\mbox{Re}}}\,(z))$.

### The average doubling time of a random gLV system

Theoretically, the doubling time of a specific species can be calculated through the intrinsic growth rate *r*, that is, Doubling time = ln2/*r*. The dynamics of a random gLV system can be represented as

$$\frac{\,{{\mbox{d}}}\,{{{\boldsymbol{X}}}}(t)}{\,{{\mbox{d}}}t}={{\mbox{diag}}}\,\left({{{\boldsymbol{X}}}}(t)\right)\left({{{\boldsymbol{r}}}}+{{{\boldsymbol{A}}}}{{{\boldsymbol{X}}}}(t)\right),$$

where ***X***(*t*) is the absolute abundance vector and ***r*** is the intrinsic growth rate vector. ***A*** is the interaction matrix, which is constructed as follows: for each pair of off-diagonal elements $\left({A}_{ij},{A}_{ji}\right)$, we draw a random value *p* from a uniform distribution $U\left[0,1\right]$. If *p* ≤ *C*, we draw *A*_*ij*_ and *A*_*ji*_ independently from a distribution with mean 0 and variance *σ*^2^. If *p* > *C*, we assign 0 to both *A*_*ij*_ and *A*_*ji*_. All diagonal elements *A*_*ii*_ are set to −1. When the system rests at a homogeneous equilibrium ${{{{\boldsymbol{X}}}}}^{\,{* }}$ = **1**, which means that ***r*** + ***A***${{{{\boldsymbol{X}}}}}^{\,{* }}$ = **0**, the intrinsic growth rate of species *i* can be derived as ${r}_{i}=1-\mathop{\sum }\nolimits_{j = 1,j\ne i}^{S}{A}_{ij}$. For a sufficiently large random community, we have ${\mathbb{E}}\left(\mathop{\sum }\nolimits_{j = 1,\,j\ne i}^{S}{A}_{ij}\right)=0$. Therefore, the average intrinsic growth rate can be obtained as $\overline{r}=1$, which leads to the average doubling time ${\overline{T}}_{\rm{d}}=\,{{\mbox{ln}}}\,2$ (see Supplementary Note 6 for more details).

### Reporting summary

Further information on research design is available in the Nature Portfolio Reporting Summary linked to this article.

### Supplementary information

Supplementary Information Supplementary Notes 1–7 and Figs. 1–22.

Reporting Summary

## Data Availability

All data analysed are simulation data and can be reproduced using the codes provided.

## Code availability

All computer codes are available at https://github.com/Pawn053/Stability-of-time-delayed-complex-ecosystems.
