# Supplementary material for: Time delays modulate the stability of complex ecosystems
Source: Nat Ecol Evol. 2023 Aug 17;7(10):1610–9. doi: 10.1038/s41559-023-02158-x (PMC10555844; doi:10.1038/s41559-023-02158-x)
Supplement: Supplementary file 1 — Supplementary Notes 1–7 and Figs. 1–22. [file 41559_2023_2158_MOESM1_ESM.pdf]

nature ecology & evolution

Article

https://doi.org/10.1038/s41559-023-02158-x

Time delays modulate the stability of complex ecosystems

In the format provided by the authors and unedited

Supplementary Information for

Time delays modulate the stability of complex ecosystems

Contents

1 Supplementary Note 1: Dynamical framework **3**

1.1 Stability analysis of delay-free ecosystems 3

1.2 Stability analysis of time-delayed ecosystems 4

2 Supplementary Note 2: Estimating the stability of complex ecosystems with time delays **7**

2.1 Stability estimation method 7

2.2 Eigenvalue distribution and stability estimation for complex interaction types 9

2.2.1 Random community 9

2.2.2 Community with complex interaction types 10

3 Supplementary Note 3: Moderate time delay stabilises the ecosystem **14**

3.1 Single species community 14

3.2 Multiple species community 16

4 Supplementary Note 4: Effect of interaction types on the stability of complex ecosystems with delay **17**

4.1 Delay-free ecosystems 17

4.2 Time-delayed ecosystems 17

5 Supplementary Note 5: Effect of species abundance on the stability of complex ecosystems with delays **19**

5.1 Delay-free ecosystems 19
5.2 Time-delayed ecosystems 19
5.2.1 Single species community 20
5.2.2 Multiple species community 20

6 Supplementary Note 6: Understanding the biological meaning of time delay intensity 21
6.1 Exponential growth and doubling time 21
6.2 Understanding the biological meaning of time delay through doubling time 21

7 Supplementary Note 7: Scaling up complexity 23
7.1 Systems with heterogeneous time delays 23
7.1.1 Model description 23
7.1.2 Stability analysis of 2-species community 24
7.1.3 Challenge in the stability analysis of large complex ecosystems 25
7.2 Toy model of bacteria competition without explicit time delays 25
7.2.1 Bacteria competition model without explicit time delays 26
7.2.2 Evaluating the influence of time delays on stability 27

1 Supplementary Note 1: Dynamical framework

In this section, we first present the canonical framework adopted by Robert May and other researchers [1–15] to introduce the stability of delay-free ecosystems. Then, we show how to analyse the stability of time-delayed scenarios.

1.1 Stability analysis of delay-free ecosystems

Similar to the framework we introduced in the main text with time delay, the delay-free ecological community composed of S interacting species can be modeled as an autonomous system described by a set of ordinary differential equations

$$\frac{\mathrm{d}\boldsymbol{X}(t)}{\mathrm{d}t} = \mathrm{diag}(\boldsymbol{X}(t))\,\boldsymbol{f}(\boldsymbol{X}(t)),$$

where $\boldsymbol{X}(t) = [X_1(t),\ X_2(t),\ \cdots,\ X_S(t)]^T$ is an S-dimensional vector whose element $X_i(t)$ represents, for example, the absolute abundance of species i at time t, $\mathrm{diag}(\boldsymbol{X}(t))$ is a square diagonal matrix with the entries of $\boldsymbol{X}(t)$ on the main diagonal and all other entries outside the main diagonal set to zero, and $\boldsymbol{f}(\boldsymbol{X}(t))$ is an unspecified function whose functional form determines the interaction structure of underlying ecological network. If $\boldsymbol{X}^* > 0$ (i.e., each species has a positive abundance) satisfies $\boldsymbol{f}(\boldsymbol{X}^*) = 0$, it is a feasible coexistence equilibrium. In ecology, we are primarily interested in the behavior of feasible coexistence equilibria, since unfeasible equilibria are those in which some species are extinct.

The dynamical behavior of an ecosystem around a feasible coexistence equilibrium can be approximated by linearising around this equilibrium

$$\frac{\mathrm{d}\boldsymbol{x}(t)}{\mathrm{d}t} \approx \mathrm{diag}(\boldsymbol{X}^*)\,\boldsymbol{J}|_{\boldsymbol{X}^*}\,\boldsymbol{x}(t). \tag{S1}$$

Here $\boldsymbol{x}(t) = \boldsymbol{X}(t) - \boldsymbol{X}^*$ captures the deviation from equilibrium abundance, each element $J_{ij}\,|_{\boldsymbol{X}^*} = \frac{\partial f_i(t)}{\partial X_j(t)}$ of $\boldsymbol{J}|_{\boldsymbol{X}^*}$ represents the per capita effect that species j has on species i around the equilibrium. For simplicity, we denote $\boldsymbol{J}|_{\boldsymbol{X}^*}$ as $\boldsymbol{J}$, and name $\boldsymbol{J}$ as 'interaction matrix'. In theoretical ecology, $\mathrm{diag}(\boldsymbol{X}^*)\,\boldsymbol{J}$ is the so-called 'community matrix', and is often denoted as $\boldsymbol{M}$. Therefore, we rewrite Eq. (S1) as

$$\frac{\mathrm{d}\boldsymbol{x}(t)}{\mathrm{d}t} = \boldsymbol{M}\boldsymbol{x}(t).$$

Here we assume that the system rests at a homogeneous equilibrium $\boldsymbol{X}^* = \mathbf{1}$ (i.e., each species has unit abundance), which brings $\boldsymbol{M} = \boldsymbol{J}$.

If all roots z of the characteristic equation

$$\det\left(z\boldsymbol{I} - \boldsymbol{M}\right) = 0,$$

have negative real parts, the corresponding equilibrium is local asymptotic stable (henceforth, stable). This leads to the stability criterion

$$\max\left(\mathrm{Re}\left(z\right)\right) < 0.$$

In fact, the eigenvalues (λ) of matrix $\boldsymbol{M}$ are the roots of the characteristic equation (i.e., $z = \lambda$). Thus, the stability criterion can be rewritten as

$$\max\left(\mathrm{Re}\left(\lambda\right)\right) < 0,$$

meaning that all eigenvalues of the community matrix should be in the left complex plane to ensure stability (Supplemental Fig. S1).

1.2 Stability analysis of time-delayed ecosystems

As we have stated in the Methods, the stability of a time-delayed system is determined by the roots of the characteristic equation [19, 22, 27]:

$$\det\left(z\boldsymbol{I} - \boldsymbol{M}\mathrm{e}^{-z\tau}\right) = 0. \tag{S2}$$

Just as the delay-free case, the equilibrium will be stable if all roots z have negative real parts, which brings the stability criterion of time-delayed systems: $\max\left(\mathrm{Re}(z)\right) < 0$. By decomposing $\boldsymbol{M}$ as $\boldsymbol{M} = \boldsymbol{P}\boldsymbol{D}\boldsymbol{P}^{-1}$ ($\boldsymbol{D}$ is the Jordan form of $\boldsymbol{M}$, and $\boldsymbol{P}$ is an invertible matrix), we have

$$\det\left(z\boldsymbol{I} - \boldsymbol{M}\mathrm{e}^{-z\tau}\right) = \det\left(\boldsymbol{P}\right)\det\left(z\boldsymbol{I} - \boldsymbol{D}\mathrm{e}^{-z\tau}\right)\det\left(\boldsymbol{P}^{-1}\right).$$

Thus, Eq. (S2) is equivalent to

$$\det\left(z\boldsymbol{I} - \boldsymbol{D}\mathrm{e}^{-z\tau}\right) = 0. \tag{S3}$$

Since $\boldsymbol{D}$ is the Jordan form of $\boldsymbol{M}$, it is an upper triangular matrix whose diagonal elements are the eigenvalues of $\boldsymbol{M}$. Eq. (S3) can be further written as

$$\prod_{i=1}^{S}\left(z-\lambda_i \mathrm{e}^{-z\tau}\right)=0,$$

where $\lambda_i \in \mathbb{C}$ is the ith eigenvalue of $\boldsymbol{M}$. Therefore, characteristic equation of time-delayed systems can also be written as $H(z)=z-\lambda\mathrm{e}^{-z\tau}=0$, as we have shown as Eq. (3) in the main text.

Different from the delay-free case, the eigenvalues of the community matrix are no longer the characteristic roots z of the characteristic equation with the presence of time delay (i.e., $z \neq \lambda$). Therefore, the stability of a time-delayed system can no longer be determined by the rightmost eigenvalue of the community matrix [19, 22, 27]. Instead, we must substitute every λ_i into the transcendental characteristic equation above to solve the corresponding z, and then check the stability. This need to check each λ_i renders determining the stability of large complex ecosystems extremely time consuming.

Then, we discuss the stability criterion of time-delayed systems from the perspective of the eigenvalue distribution of the community matrix. Previous studies [19, 22, 27] suggested that the sign change (from a negative value to positive value) of $\mathrm{Re}(z)$ occurs when $z=i\cdot\omega,\ \omega\in\mathbb{R}$. Due to the symmetry of z, we only need to consider nonnegative ω, that is, $z=i\cdot\omega,\ \omega\in\mathbb{R}_0^+$. Substituting $z=i\cdot\omega$ into the characteristic equation, we have

$$i\cdot\omega-(x+i\cdot y)\,\mathrm{e}^{-i\cdot\omega\tau}=0,$$

where $x=\mathrm{Re}(\lambda),\ y=\mathrm{Im}(\lambda)$. The above equation can be further written as

$$\begin{cases} x\cos(\omega\tau)+y\sin(\omega\tau)=0, \\ -x\sin(\omega\tau)+y\cos(\omega\tau)=\omega. \end{cases} \tag{S4}$$

By solving Eq. (S4), we obtain the boundary of λ (Fig. 1f and Supplemental Fig. S1)

$$\tau=\frac{1}{\sqrt{x^2+y^2}}\tan^{-1}\left(-\frac{x}{y}\right).$$

This boundary encloses a teardrop-shaped region. For an ecosystem with time delay τ, stability requires that the eigenvalues of its community matrix must locate in this region. Thus, the introduc-

tion of time delays imposes more strict requirements to the eigenvalue distribution: the eigenvalues of the community matrix should not only distribute in the left half complex plane, but also locate in the corresponding teardrop-shaped region. Again, we emphasise that to determine the stability of a time-delayed ecosystem, one can no longer simply focus on the rightmost eigenvalue but must instead consider the whole eigenvalue distribution. We also notice that, as τ increases, this stability region shrinks (Supplemental Fig. S1). These observations suggest that the introduction of time delays can induce instability, as suggested by previous work [1, 19, 22]. Thus, time delays play an important role in shaping ecosystem stability.

2 Supplementary Note 2: Estimating the stability of complex ecosystems with time delays

Discussion in Supplementary Note 1 shows that the stability of time-delayed ecosystems is determined by $-\max(\mathrm{Re}(z))$. Thus, the typical stability analysis procedure of time-delayed systems includes following steps:

Step 1. Calculate the eigenvalues (λ) of the community matrix $\boldsymbol{M}$.

Step 2. Substitute each eigenvalue λ_i into the characteristic equation and calculate the corresponding z.

Step 3. Compare all z obtained in step 2 and find $-\max(\mathrm{Re}(z))$.

Due to the presence of the exponential term, Eq. (3) is a transcendental equation. Therefore, stability analysis of multiple species ecosystems with time delay is a non-deterministic polynomial (NP)-hard problem [36]. That is, stability analysis of time-delayed ecosystems is extremely time-consuming when S is large. This NP-hard nature may be the reason why most existing works on time-delayed ecosystems are limited to low diversity communities. Since single species models have only one real eigenvalue, the range of delay lengths leading to a stable community can be theoretically obtained (as is presented in Supplementary Note 3), meaning determining whether or not a single species community is stable is not an NP-hard problem.

In this section, we solve this problem by first identifying the unique pattern of stability region, and then combining this with observations from random matrix theory and low-rank perturbations theorem [4, 7, 9, 13, 43, 44].

2.1 Stability estimation method

In Supplementary Note 1, we identify the stability region in the complex plane by solving the equation with $z = i \cdot \omega,\ \omega \in \mathbb{R}$. Here we let $z = -\alpha + i \cdot \omega,\ \alpha \in \mathbb{R}_0^+,\ \omega \in \mathbb{R}$, and solve the characteristic equation. By doing so, we are able to find a stability region with minimum level of stability α (henceforth, α-stability region) in the complex plane.

Due to the symmetry of z, we only need to consider nonnegative ω, that is, $\omega \in \mathbb{R}_0^+$. Substituting

$z = -\alpha + i\cdot\omega,\ \alpha \in \mathbb{R}_0^+,\ \omega \in \mathbb{R}_0^+$ into Eq. (3), we have

$$-\alpha + i\cdot\omega - (x + i\cdot y)\,\mathrm{e}^{-(-\alpha+i\cdot\omega)\tau} = 0,$$

which brings the boundary of the α-stability region

$$\begin{cases} \tau = \frac{1}{\omega}\tan^{-1}\left(\frac{-\alpha y-\omega x}{-\alpha x+\omega y}\right), \\ \omega = \sqrt{\mathrm{e}^{2\alpha\tau}\left(x^2+y^2\right)-\alpha^2}. \end{cases}$$

By increasing α from 0 and plotting the corresponding boundary, we obtain the stability contour plot in the complex plane (Supplemental Fig. S2). We first notice that each α-stability region is still a teardrop-shaped region. As α increases, the corresponding teardrop-shaped region shrinks to the center from all directions (this means that the boundaries of different α-stability regions are non-crossing). Thus, the closer an eigenvalue is to the boundary of the stability region, the smaller the corresponding $-\mathrm{Re}(z)$ value (i.e., lower level of stability).

Next we note that for large complex ecosystems, the eigenvalues of the community matrix typically distribute in a circle or an ellipse (sometimes with an outlier) [4,7,9]. Therefore, the four endpoints of the eigenvalue distribution are more likely to locate near the boundary of the stability region (Fig. 4). As such, to estimate the stability of a time-delayed system, we need only consider the four endpoints of the eigenvalue distribution among all eigenvalues. Moreover, since the eigenvalue distribution of $\boldsymbol{M}$ and the stability region are all symmetric about the real axis, the uppermost eigenvalue and the lowermost eigenvalue are equivalent. Hence, three endpoints (rightmost, leftmost, and uppermost) are often sufficient to define the corresponding community stability. Crucially, each of these three eigenvalues can be analytically obtained through random matrix theory and low-rank perturbation theorem. Thus, we now only need to solve the characteristic equation three times instead of S times to get the estimation of stability. This greatly reduces the time cost for stability analysis, and provides a possible way to deal with this NP-hard problem.

Our stability estimation method is thus simply defined as:

Step 1. Find three endpoints (rightmost, leftmost and uppermost) of the eigenvalue distribution of $\boldsymbol{M}$.

Step 2. Substitute these three endpoints into the characteristic equation and calculate the corresponding z.

Step 3. Compare all z obtained in step 2 and find the estimation for $-\max(\mathrm{Re}(z))$.

2.2 Eigenvalue distribution and stability estimation for complex interaction types

Since we now only need to consider three endpoints of the eigenvalue distribution to estimate stability, the next problem is to find these endpoints. In this section, we show how to estimate the stability of random communities and communities with complex interaction types. For simplicity, we denote three statistical features of community matrix by $\mathbb{E}(M_{ij,i\neq j}) = E$, $\mathrm{Var}(M_{ij,i\neq j}) = V$ and $\mathbb{E}(M_{ij,i\neq j}M_{ji,i\neq j}) = \rho$. Note that due to our assumptions about community equilibria, $\boldsymbol{M} = \boldsymbol{J}$, thus these statistical features can be extracted directly from the corresponding interaction matrix.

2.2.1 Random community

From the construction of the interaction matrix and the assumption of equilibrium (see Methods in main text), we can obtain $\mathbb{E}(M_{ij,i\neq j}) = E = 0$, $\mathrm{Var}(M_{ij,i\neq j}) = V = C\sigma^2$, and $\mathbb{E}(M_{ij,i\neq j}M_{ji,i\neq j}) = \rho = 0$. Here we first consider the eigenvalue distribution of matrix $\boldsymbol{M'} = \boldsymbol{M} + s\boldsymbol{I}$. The mean of the elements of $\boldsymbol{M'}$ is $\mathbb{E}\left(M'_{ij}\right) = 0$, and the variance of the elements of $\boldsymbol{M'}$ is $\mathrm{Var}\left(M'_{ij}\right) = C\sigma^2$. Then we consider matrix $\boldsymbol{N} = \boldsymbol{M'}/\sqrt{S\cdot\mathrm{Var}\left(M'_{ij}\right)}$. We can quickly find that the mean of the elements of $\boldsymbol{N}$ is $\mathbb{E}(N_{ij}) = 0$ and the variance of the elements of $\boldsymbol{N}$ is $\mathrm{Var}(N_{ij}) = 1/S$. Thus, $\boldsymbol{N}$ satisfies the circular law [4, 7, 9, 45]. As $S\to\infty$, the eigenvalues of $\boldsymbol{N}$ are uniformly distributed in a unit disk centered at $(0,0)$. This indicates that when S is sufficiently large, the eigenvalue distribution of $\boldsymbol{M'}$ is uniform on a circle of radius approximately $\sigma\sqrt{SC}$ centered at $(0,0)$.

Now we turn to matrix $\boldsymbol{M} = \boldsymbol{M'} - s\boldsymbol{I}$, which introduces the effect of diagonal elements. Since all diagonal entries are $-s$, the eigenvalue distribution of $\boldsymbol{M}$ is shifted leftwards and centered at $(-s,0)$ compared with $\boldsymbol{M'}$. Thus, when S is sufficiently large, the eigenvalues of $\boldsymbol{M}$ are uniformly distributed in a circle with radius approximately $\sigma\sqrt{SC}$ centered at $(-s,0)$. Three endpoints (leftmost, rightmost, and uppermost) of the distribution can then be estimated as

$$\begin{cases} Q_{\text{leftmost}}\left(-s-\sigma\sqrt{SC},0\right), \\ Q_{\text{rightmost}}\left(-s+\sigma\sqrt{SC},0\right), \\ Q_{\text{uppermost}}\left(-s,\sigma\sqrt{SC}\right). \end{cases}$$

2.2.2 Community with complex interaction types

Based on the signs of M_{ij}/M_{ji}, there are three typical types of interactions [4, 9], namely, +/+ (mutualism), -/- (competition), +/- (exploitative). From the construction of interaction matrix (see Methods in the main text), we have the mean, variance and correlation of the elements of $\boldsymbol{M}$

$$\begin{cases} E = \mathbb{E}\left(M_{ij,i\neq j}\right) = C\mathbb{E}\left(|Z|\right)\left(P_{\mathrm{m}} - P_{\mathrm{c}}\right), \\ V = \mathrm{Var}\left(M_{ij,i\neq j}\right) = \mathbb{E}\left(M_{ij,i\neq j}^2\right) - \mathbb{E}\left(M_{ij,i\neq j}\right)^2, \\ \rho = \mathbb{E}\left(M_{ij,i\neq j}M_{ji,i\neq j}\right) = C\mathbb{E}^2(|Z|)\left(P_{\mathrm{m}} + P_{\mathrm{c}} - P_{\mathrm{e}}\right), \end{cases}$$

where P_{m} is the proportion of mutualistic interactions, P_{c} is the proportion of competitive interactions, and P_e is the proportion of exploitative interactions. Since $\mathbb{E}\left(M_{ij,i\neq j}^2\right) = C\mathbb{E}\left(|Z|^2\right)\left(P_{\mathrm{m}} + P_{\mathrm{c}} + P_{\mathrm{e}}\right)$, the equation above can be rewritten as

$$\begin{cases} E = \mathbb{E}\left(M_{ij,i\neq j}\right) = C\mathbb{E}(|Z|)\left(P_{\mathrm{m}} - P_{\mathrm{c}}\right), \\ V = \mathrm{Var}\left(M_{ij,i\neq j}\right) = C\sigma^2 - E^2, \\ \rho = \mathbb{E}\left(M_{ij,i\neq j}M_{ji,i\neq j}\right) = C\mathbb{E}^2\left(|Z|\right)\left(P_{\mathrm{m}} + P_{\mathrm{c}} - P_{\mathrm{e}}\right). \end{cases}$$

Just as the random case, we first consider matrix $\boldsymbol{M'} = \boldsymbol{M} + s\boldsymbol{I}$ with

$$\begin{cases} \mathbb{E}\left(M'_{ij}\right) = E, \\ \mathrm{Var}\left(M'_{ij}\right) = V, \\ \mathbb{E}\left(M'_{ij}M'_{ji}\right) = \rho. \end{cases}$$

Since $\mathbb{E}\left(M'_{ij}\right) \neq 0$, the eigenvalue distribution cannot be obtained by the procedures used in the random case (that is, rescale the matrix and apply random matrix theory directly). Instead, we rewrite $\boldsymbol{M'}$ as

$$\begin{aligned} \boldsymbol{M'} &= \left(\boldsymbol{M'} - E\cdot\mathbf{1}\cdot\mathbf{1}^{\mathrm{T}} + E\boldsymbol{I}\right) + E\cdot\mathbf{1}\cdot\mathbf{1}^{\mathrm{T}} - E\boldsymbol{I} \\ &= \boldsymbol{N} + E\cdot\mathbf{1}\cdot\mathbf{1}^{\mathrm{T}} - E\boldsymbol{I}, \end{aligned}$$

where $\mathbf{1}\cdot\mathbf{1}^{\mathrm{T}}$ is an $S\times S$ matrix with each entry equal to 1. We then have $N_{ij,i\neq j} = M'_{ij,i\neq j} - \mathrm{E}$, $N_{ii} =$

$M'_{ii} = 0$. The corresponding statistical features of $\boldsymbol{N}$ are now

$$\begin{cases} \mathbb{E}\left(N_{ij}\right) = \mathbb{E}\left(M'_{ij} - E\right) = 0, \\ \mathrm{Var}\left(N_{ij}\right) = \mathrm{Var}\left(M'_{ij}\right) = V, \\ \mathbb{E}\left(N_{ij}N_{ji}\right) = \mathbb{E}\left(\left(M'_{ij} - E\right)\left(M'_{ji} - E\right)\right) = \rho - E^2. \end{cases}$$

It is clear that $\boldsymbol{N}$ is now an elliptic matrix. We then construct matrix $\boldsymbol{N}^* = \boldsymbol{N}/\beta$, where $\beta = \sqrt{S \cdot \mathrm{Var}\left(N_{ij}\right)}$. The mean, variance and correlation of $\boldsymbol{N}^*$ are thus

$$\begin{cases} \mathbb{E}\left(N^*_{ij}\right) = 0, \\ \mathrm{Var}\left(N^*_{ij}\right) = \frac{1}{S}, \\ \mathbb{E}\left(N^*_{ij}N^*_{ji}\right) = \frac{\rho - E^2}{\beta^2}. \end{cases}$$

$\boldsymbol{N}^*$ is then a standard elliptic matrix [4,9,43]. Thus, for large S, the eigenvalues of $\boldsymbol{N}^*$ are uniformly distributed in an ellipse

$$\left(\frac{x}{a_{\boldsymbol{N}^*}}\right)^2 + \left(\frac{y}{b_{\boldsymbol{N}^*}}\right)^2 \leq 1,$$

where $a_{\boldsymbol{N}^*} = 1 + r$, $b_{\boldsymbol{N}^*} = 1 - r$, and $r = \left(\rho - E^2\right)/V$. Therefore, we know eigenvalues of $\boldsymbol{N}$ distribute in an ellipse

$$\left(\frac{x}{a_{\boldsymbol{N}}}\right)^2 + \left(\frac{y}{b_{\boldsymbol{N}}}\right)^2 \leq 1,$$

with $a_{\boldsymbol{N}} = \beta\left(1 + r\right)$ and $b_{\boldsymbol{N}} = \beta\left(1 - r\right)$.

Turning to $\boldsymbol{M'} = \boldsymbol{N} + E \cdot \boldsymbol{1} \cdot \boldsymbol{1}^{\mathrm{T}} - E\boldsymbol{I}$, we have that $E \cdot \boldsymbol{1} \cdot \boldsymbol{1}^{\mathrm{T}}$ is a rank-one perturbation: $S-1$ eigenvalues are zero, and a single eigenvalue is SE. According to the low-rank perturbation theorem [44], when $|E| < \sqrt{\mathrm{Var}(N_{ij})/S}$ (i.e, $|E| < \sqrt{V/S}$), eigenvalues of $\boldsymbol{N} + \mathrm{E} \cdot \boldsymbol{1} \cdot \boldsymbol{1}^T$ still distribute in the ellipse

$$\left(\frac{x}{a_{\boldsymbol{N}}}\right)^2 + \left(\frac{y}{b_{\boldsymbol{N}}}\right)^2 \leq 1.$$

When $|E| > \sqrt{\mathrm{Var}(N_{ij})/S}$ (i.e, $|E| > \sqrt{V/S}$), $S - 1$ eigenvalues of $\boldsymbol{N} + \mathrm{E} \cdot \boldsymbol{1} \cdot \boldsymbol{1}^{\mathrm{T}}$ distribute in the ellipse above, while an eigenvalue is modified. This single eigenvalue is approximately equal to $SE + \left(\rho - E^2\right)/E$. When S is sufficiently large, this single eigenvalue can be further simplified to $SE = SC\mathbb{E}(|Z|)\left(P_m - P_c\right)$. Finally, the effect of $-E\boldsymbol{I}$ is to shift the whole distribution by substracting E from each eigenvalue. Therefore, for sufficiently large S, we obtain the eigenvalue distribution of $\boldsymbol{M'}$:

When $|E| < \sqrt{V/S}$:

$$\left(\frac{x+E}{a_{\boldsymbol{M}'}}\right)^2 + \left(\frac{y}{b_{\boldsymbol{M}'}}\right)^2 \leq 1,$$

with $a_{\boldsymbol{M}'} = \beta(1+r)$ and $b_{\boldsymbol{M}'} = \beta(1-r)$.

When $|E| > \sqrt{V/S}$:

$$\begin{cases} \left(\frac{x+E}{a_{\boldsymbol{M}'}}\right)^2 + \left(\frac{y}{b_{\boldsymbol{M}'}}\right)^2 \leq 1, \\ \lambda_{\boldsymbol{M}',\text{outlier}} = (S-1)E. \end{cases}$$

Finally, by introducing the effect of diagonal elements, we derive the eigenvalue distribution of $\boldsymbol{M}$,

$$\begin{cases} \left(\frac{x+E+s}{a}\right)^2 + \left(\frac{y}{b}\right)^2 \leq 1, \\ \lambda_{\text{outlier}} = -s + (S-1)E, \ (\text{when } |E| > \sqrt{\frac{V}{S}}), \end{cases}$$

where $a = \beta(1+r)$ and $b = \beta(1-r)$. The three endpoints of the eigenvalue distribution can therefore be estimated as,

When $|E| < \sqrt{\mathrm{V}/S}$:

$$\begin{cases} Q_{\text{leftmost}}\left(c-a, 0\right), \\ Q_{\text{rightmost}}\left(c+a, 0\right), \\ Q_{\text{uppermost}}\left(c, b\right), \end{cases}$$

where $c = -E - s$.

When $|E| > \sqrt{\mathrm{V}/S}$:

$$\begin{cases} Q_{\text{leftmost}}\left(\min\left(c-a, \lambda_{\text{outlier}}\right), 0\right), \\ Q_{\text{rightmost}}\left(\max\left(c+a, \lambda_{\text{outlier}}\right), 0\right), \\ Q_{\text{uppermost}}\left(c, b\right). \end{cases}$$

With this general definition in hand, we can then estimate the eigenvalue distributions of four typical communities as:

(1) Exploitative community ($P_{\text{e}} = 1$)

$$\begin{cases} Q_{\text{leftmost}}\left(-s - \sigma\sqrt{SC}\left(1 - \frac{\mathbb{E}^2(|Z|)}{\sigma^2}\right), 0\right), \\ Q_{\text{rightmost}}\left(-s + \sigma\sqrt{SC}\left(1 - \frac{\mathbb{E}^2(|Z|)}{\sigma^2}\right), 0\right), \\ Q_{\text{uppermost}}\left(-s, \sigma\sqrt{SC}\left(1 + \frac{\mathbb{E}^2(|Z|)}{\sigma^2}\right)\right). \end{cases}$$

(2) Community with mixed interactions of competition and mutualism ($P_{\mathrm{c}} = P_{\mathrm{m}} = 0.5$)

$$\begin{cases} Q_{\text{leftmost}}\left(-s-\sigma\sqrt{SC}\left(1+\frac{\mathbb{E}^2(|Z|)}{\sigma^2}\right),0\right), \\ Q_{\text{rightmost}}\left(-s+\sigma\sqrt{SC}\left(1+\frac{\mathbb{E}^2(|Z|)}{\sigma^2}\right),0\right), \\ Q_{\text{uppermost}}\left(-s,\sigma\sqrt{SC}\left(1-\frac{\mathbb{E}^2(|Z|)}{\sigma^2}\right)\right). \end{cases}$$

(3) Competitive community ($P_{\mathrm{c}} = 1$)

$$\begin{cases} Q_{\text{leftmost}}\left(-s-\left(S-1\right)C\mathbb{E}(|Z|),0\right), \\ Q_{\text{rightmost}}\left(-s+C\mathbb{E}(|Z|)+\sqrt{SC(\sigma^2-C\mathbb{E}^2(|Z|))}\frac{\sigma^2+(1-2C)\mathbb{E}^2(|Z|)}{\sigma^2-C\mathbb{E}^2(|Z|)},0\right), \\ Q_{\text{uppermost}}\left(-s+C\mathbb{E}(|Z|),\sqrt{SC(\sigma^2-C\mathbb{E}^2(|Z|))}\frac{\sigma^2-\mathbb{E}^2(|Z|)}{\sigma^2-C\mathbb{E}^2(|Z|)}\right). \end{cases}$$

(4) Mutualistic community ($P_{\mathrm{m}} = 1$)

$$\begin{cases} Q_{\text{leftmost}}\left(-s-C\mathbb{E}(|Z|)-\sqrt{SC(\sigma^2-C\mathbb{E}^2(|Z|))}\frac{\sigma^2+(1-2C)\mathbb{E}^2(|Z|)}{\sigma^2-C\mathbb{E}^2(|Z|)},0\right), \\ Q_{\text{rightmost}}\left(-s+\left(S-1\right)C\mathbb{E}(|Z|),0\right), \\ Q_{\text{uppermost}}\left(-s-C\mathbb{E}(|Z|),\sqrt{SC\left(\sigma^2-C\mathbb{E}^2\left(|Z|\right)\right)}\frac{\sigma^2-\mathbb{E}^2(|Z|)}{\sigma^2-C\mathbb{E}^2(|Z|)}\right). \end{cases}$$

By substituting the three endpoints (rightmost, leftmost, and uppermost) derived in this section into the characteristic equation, we can then get an estimation of stability. Importantly, results from numerical simulations are in good agreement with our theoretical estimations, which prove the validity of our approach developed here (Supplemental Figs. S3, S6, S7, S11).

3 Supplementary Note 3: Moderate time delay stabilises the ecosystem

In this section, we explore the influence of the intensity of time delay on the stability of ecosystems. We first assess this influence on a single species community and then discuss the case of large complex ecosystems.

3.1 Single species community

For a single species community, the linearised equation becomes

$$\frac{\mathrm{d}x\left(t\right)}{\mathrm{d}t} = -sX^{*}x\left(t-\tau\right) = -sx\left(t-\tau\right),$$

where $X^{*} = 1$. The only eigenvalue of the community matrix of this single species community is $\lambda = -s$, which brings the characteristic equation

$$H(z) = z + s\mathrm{e}^{-\tau z} = 0. \tag{S5}$$

In this section, we perform a theoretical analysis for this single species community to complement the intuitive analysis in the main text.

To perform the theoretical analysis, we first introduce the concept of Lambert W function [46–49]. When the function satisfies

$$W(a)\mathrm{e}^{W(a)} = a,$$

it is defined as the Lambert W function. Lambert W function is a multi-valued function, meaning that it has infinite number of branches. These branches can be expressed as $W_k(a),\ k = 0,\ \pm1,\ \cdots,\ \pm\infty$, and are all single-valued functions. Especially, $W_0(a)$ is said to be the principal branch. An important property [47] of $W_0(a)$ is that it is a real-valued monotone increasing function when $a \in [-1/\mathrm{e},\ \infty)$.

With the help of Lambert W function, the infinite number of solutions of Eq. (S5) can be expressed as

$$z_i = \frac{1}{\tau}W_i(-\tau s),\ i = 0, \cdots, \infty.$$

To draw the relationship between stability and time delay intensity, we give following lemmas:

Lemma 1. For arbitrary $a \in \mathbb{C}$, $\max\{\mathrm{Re}(W_k(a))|k = 0,\ \pm1,\ \cdots,\ \pm\infty\} = \mathrm{Re}(W_0(a))$ holds.

Proof. See the work by Shinozaki and Mori for the proof [48].

Remark. From Lemma 1, it is clear that the stability of the single species community is determined by the characteristic root corresponding to the principal branch of the Lambert W function, i.e., $\max(\mathrm{Re}(z)) = \max(\mathrm{Re}(W_0(-\tau s)/\tau))$. Stability can then be represented as Stability $= -\max(\mathrm{Re}(W_0(-\tau s)/\tau))$. Since $W_0(a)$ is a real-valued monotonically increasing function when $a \in [-1/\mathrm{e},\ \infty)$, stability increases as τ increases from 0 to $1/(s\mathrm{e})$.

Lemma 2. Eq. (S5) has no real roots when $\tau s\mathrm{e} > 1$ [22].

Proof. Suppose Eq. (S5) has a real root $z = -\mu$ when $\tau s\mathrm{e} > 1$. We then have $\mu = s\mathrm{e}^{\tau\mu}$. It is clear that $\mu > 0$ (since $s > 0,\ \mathrm{e}^{\tau\mu} > 0$). From $\mu = s\mathrm{e}^{\tau\mu}$, we can draw that $1 = s\tau(\mathrm{e}^{\tau\mu}/(\tau\mu))$. Let $g(x) = \mathrm{e}^x - \mathrm{e}x,\ x \in (0,\ +\infty)$. We can prove that $g(x)_{\min} = g(1) = 0$. Thus, $\mathrm{e}^x/x \geq \mathrm{e}$ holds when $x \in (0,\ +\infty)$. Since $\mu > 0$, we have $\mathrm{e}^{\tau\mu}/(\tau\mu) \geq \mathrm{e}$, and therefore, $s\tau(\mathrm{e}^{\tau\mu}/(\tau\mu)) \geq \tau s\mathrm{e}$. Since $1 = s\tau(\mathrm{e}^{\tau\mu}/(\tau\mu))$, we have $\tau s\mathrm{e} \leq 1$, which contradicts the relation $\tau s\mathrm{e} > 1$. Therefore, we arrive at the conclusion that when $\tau s\mathrm{e} > 1$ holds, Eq. (S5) have no real roots, which completes the proof of Lemma 2.

Lemma 3. The single species community depicted by Eq. (S5) becomes unstable when $\tau \geq \tau_{\max} = \pi/(2s)$.

Proof. As the single species community has only one real eigenvalue $\lambda_1 = -s$, and the right endpoint and left endpoint of the teardrop-shaped stability region can be theoretically obtained as $Q_{\text{left}}(-\pi/(2\tau), 0)$ and $Q_{\text{right}}(0, 0)$, then if this single eigenvalue locates between these two endpoints, the system is stable. That is, as long as time delay $\tau < -\pi/(2\lambda_1) = \pi/(2s)$, the system is stable. Other forms for the proof of this lemma are reported in references like [19] and [22].

Remark. In the field of system and control, this $\tau_{\max}$ is the so-called 'delay margin' [33].

Lemma 4. For the roots of Eq. (S5) satisfying $\mathrm{Re}(z) < 0$ and $\mathrm{Im}(z) \neq 0$, $\mathrm{Re}(z)$ increases as τ increases.

Proof. Suppose $z = x_z + i \cdot y_z$ ($x_z \in \mathbb{R}^-,\ y_z \in \mathbb{R}$ and $y_z \neq 0$) and substitute z into Eq. (S5), we have

$$\ln\left(\frac{1}{s}\sqrt{x_z^2 + y_z^2}\right) + i \cdot \left(\tan^{-1}\left(\frac{y_z}{x_z}\right) + 2k\pi\right) = -\left(x_z + i \cdot y_z\right)\tau,\ (k \in \mathbb{Z}).$$

This equation is equivalent to

$$\begin{cases} -\tau x_z = \ln\left(\frac{1}{s}\sqrt{x_z^2+y_z^2}\right), \\ -\tau y_z = \tan^{-1}\left(\frac{y_z}{x_z}\right) + 2k\pi. \end{cases}$$

y_z can then be expressed as a function of τ and x_z: $y_z = \pm\sqrt{s^2 \mathrm{e}^{-2\tau x_z} - x_z^2}$. Due to the conjugation of roots, we only need to consider $y_z = \sqrt{s^2 \mathrm{e}^{-2\tau x_z} - x_z^2}$. Suppose $g(\tau, x_z) = \sqrt{s^2 \mathrm{e}^{-2\tau x_z} - x_z^2}$, we have

$$\begin{cases} \frac{\partial g}{\partial \tau} = -\frac{2x_z^2 - s^2 \mathrm{e}^{-2\tau x_z} + s^2 \tau x_z \mathrm{e}^{-2\tau x_z}}{\sqrt{s^2 \mathrm{e}^{-2\tau x_z} - x_z^2}}, \\ \frac{\partial g}{\partial x_z} = -\frac{2\tau x_z + s^2 \tau^2 \mathrm{e}^{-2\tau x_z} + 1}{\sqrt{s^2 \mathrm{e}^{-2\tau x_z} - x_z^2}}. \end{cases}$$

And thus

$$\frac{\mathrm{d}x_z}{\mathrm{d}\tau} = \frac{2x_z^2 - s^2 \mathrm{e}^{-2\tau x_z} + s^2 \tau x_z \mathrm{e}^{-2\tau x_z}}{2\tau x_z + s^2 \tau^2 \mathrm{e}^{-2\tau x_z} + 1}.$$

For the denominator of equation above, we can prove that $2\tau x_z + s^2\tau^2 \mathrm{e}^{-2\tau x_z} + 1 \geq 2\tau x_z + \tau^2 x_z^2 + 1 \geq 0$ (since $y_z \geq 0$). For the nominator of equation above, let $h(\tau) = 2x_z^2 - s^2 \mathrm{e}^{-2\tau x_z} + s^2 \tau x_z \mathrm{e}^{-2\tau x_z}$. Since $\mathrm{d}h/\mathrm{d}\tau = s^2 \mathrm{e}^{-2\tau x_z (-2\tau x_z^2 + 3x_z)} < 0$, $h(\tau) > h(\tau_{\max}) = h(\pi/(2s)) = 0$ (Lemma 3). Thus, $\mathrm{d}x_z/\mathrm{d}\tau > 0$ and the proof of Lemma 4 is completed.

Based on Lemma 1 to Lemma 4, we prove that as the intensity of time delay increases, stability first increases and then decreases. Specifically, when $\tau \in [0, 1/(s\mathrm{e}))$, stability increases as time delay increases (Lemma 1). When $\tau \in [1/(s\mathrm{e}), \pi/(2s))$, stability decreases as time delay increases (Lemma 2 and Lemma 4). When $\tau > \pi/(2s)$, the system is unstable (Lemma 3). Stability peaks at $\tau = 1/(s\mathrm{e})$, and this peak can be theoretically obtained: $\text{Stability}_{\max} = s\mathrm{e}$. These conclusions are further confirmed by numerical calculations (Supplemental Fig. S4).

3.2 Multiple species community

With the stability analysis method developed in Supplementary Note 2, we are able to explore the influence of the intensity of time delay on large complex ecosystems. Our results show that this non-monotonic relationship between time delay and stability also establishes in multiple species communities, independent of diversity, complexity, or interaction types (Fig. 4a and Supplemental Figs. S6, S7).

4 Supplementary Note 4: Effect of interaction types on the stability of complex ecosystems with delay

The stability estimation method developed in Supplementary Note 2 also allows us to evaluate the influence of different interaction types on stability. In this section, we first briefly review this influence on delay-free systems, which is studied by the seminal work of Allesina & Tang [4] and Coyte, Schulter & Foster [9]. Then, we show that the scenario is dramatically different in time-delayed systems.

4.1 Delay-free ecosystems

With the eigenvalue distributions derived in Supplementary Note 2, the stability of different typical communities can then be obtained [4, 9]

$$\begin{cases} \text{Stability}_{\text{random}} = s - \sigma\sqrt{SC}, \\ \text{Stability}_{+/-} = s - \sigma\sqrt{SC}\left(1 - \frac{\mathbb{E}^2(|Z|)}{\sigma^2}\right), \\ \text{Stability}_{-/-} = s + C\mathbb{E}(|Z|) - \sqrt{SC\left(\sigma^2 - C\mathbb{E}^2\left(|Z|\right)\right)} \cdot \frac{\sigma^2 + (1-2C)\mathbb{E}^2(|Z|)}{\sigma^2 - C\mathbb{E}^2(|Z|)}, \\ \text{Stability}_{+/+} = s - (S-1)\, C\mathbb{E}\left(|Z|\right). \end{cases}$$

An important observation from equations above is that the stability of these 4 classic communities forms a strict hierarchy

$$\text{Stability}_{\text{exploitative}} > \text{Stability}_{\text{random}} > \text{Stability}_{\text{competition}} > \text{Stability}_{\text{mutualism}}.$$

This is the result from Allesina & Tang's seminal work [4], suggesting that exploitative communities are most likely to be stable. Further work by Coyte *et al.* studied the stability of mixed delay-free communities [9]. They found that the increase of mutualistic interactions in competitive communities is detrimental to stability.

4.2 Time-delayed ecosystems

We then show that when the time delay is considered, the previous two conclusions are deeply changed. To explore the stability performance of ecosystems with time delays, we depict the contour plots of stability under different time delays (Fig. 4 and Supplemental Fig. S8). When time delay is small, the curvature of these contour lines is relatively small and the contour plots are like those of the delay-free case. Thus, the stability of different types of communities is still determined by the rightmost eigen-

value, and exploitative community possesses the best stability performance (Fig. 4 and Supplemental Fig. S10). As time delay increases, the curvatures of these contour lines increase and the stability region shrinks, and the rightmost eigenvalue then can't determine the stability.

For exploitative communities, the eigenvalues of the community matrix are uniformly distributed in a vertically stretched ellipse when compared with random communities (Fig. 4c and Supplemental Fig. S9). This vertical distortion makes the exploitative communities more likely to reach the low stability area from the top (and below). Thus, random communities are more stable than exploitative communities.

For competitive communities, the eigenvalues of the community matrix are divided into two parts: the bulk of eigenvalues are uniformly distributed in a horizontally stretched ellipse, and an outlier on the left of this ellipse (Fig. 4c and Supplemental Fig. S9). Compared with random communities, this horizontal stretch distorts the eigenvalue distribution and the outlier aggravates the distortion. Thus, competitive communities are more likely to reach the low stability area from the left (or right) than random communities. This makes competitive communities less stable than random communities.

For mutualistic communities, the eigenvalue distribution is just like that of competitive communities but the outlier is located on the right of the ellipse. Therefore, mutualistic communities are also more likely to reach the low stability area from the right (or left) when compared with random communities (Fig. 4c and Supplemental Fig. S9). Random communities are then more stable than mutualistic communities.

We then conclude that when the time delay is sufficiently large, random communities show the best stability performance among these classic communities, which is different from the delay-free case (Fig. 4a and Supplemental Fig. S10).

Further analysis on communities with mixed interactions of exploitation, competition and mutualism shows that communities with diverse interaction types are more stable than communities with a single type of interactions (Supplemental Fig. S11). We then arrive at the conclusion that a diversity of interaction types can lead to a more stable community with the presence of time delays.

5 Supplementary Note 5: Effect of species abundance on the stability of complex ecosystems with delays

In former Supplementary Notes, we study the stability of large complex ecosystems with time delays by assuming that the community rests at a homogeneous equilibrium $\boldsymbol{X}^* = \mathbf{1}$. It is believed that species equilibrium abundance distribution has an important influence on ecosystem stability [14], and an intuitive view point is that higher species abundance can lead to a more stable community [38–40]. Recently, results from delay-free cases showed that equilibrium abundance distribution does not affect the stability qualitatively [14]. We then wonder how the equilibrium abundance shapes the stability of time-delayed systems.

While keeping matrix $\boldsymbol{J}$ constant, we vary species equilibrium abundance to study the stability of time-delayed ecosystems. For simplicity, we assume that the system still rests at a homogeneous equilibrium, meaning that all species have the same equilibrium abundance (i.e., $X_i^* = X^*$). Note that now we have

$$\boldsymbol{M} = \mathrm{diag}(\boldsymbol{X}^*)\boldsymbol{J} = X^*\boldsymbol{J}.$$

5.1 Delay-free ecosystems

The fact that $\boldsymbol{M} = X^*\boldsymbol{J}$ leads to the following relationship between the eigenvalues (λ) of $\boldsymbol{M}$ and the eigenvalues ($\lambda_{\boldsymbol{J}}$) of $\boldsymbol{J}$

$$\lambda_i = X^*\lambda_{\boldsymbol{J},i}.$$

Therefore, we have $\max(\mathrm{Re}(\lambda)) = X^* \cdot \max(\mathrm{Re}(\lambda_{\boldsymbol{J}}))$. Feasibility requires that $X^* > 0$, and thus species abundance doesn't change the sign of $\max(\mathrm{Re}(\lambda_{\boldsymbol{J}}))$. We can then recover the conclusion that species equilibrium abundances do not influence stability qualitatively: as long as $\max(\mathrm{Re}(\lambda_{\boldsymbol{J}})) < 0$, $\max(\mathrm{Re}(\lambda)) < 0$. However, it does change the stability level (Supplemental Figs. S12, S13): for stable delay-free ecosystems (i.e., $\max(\mathrm{Re}(\lambda_{\boldsymbol{J}})) < 0$), the increase of species abundance X^* makes the system more stable (Fig. 5a, Supplemental Figs. S12, S13).

5.2 Time-delayed ecosystems

Next, we consider time-delayed systems. As previously, here we first discuss the theoretical analysis of a single species community. Then, with the help of the stability estimation framework developed in Supplementary Note 2, we extend the discussion to multiple species communities.

5.2.1 Single species community

For a single species community, the linearised equation becomes

$$\frac{\mathrm{d}x(t)}{\mathrm{d}t} = -X^* s x(t-\tau).$$

Since the eigenvalue of the community matrix is $\lambda = -X^* s$, the characteristic equation becomes

$$H(z) = z + X^* s \mathrm{e}^{-z\tau} = 0.$$

It is then clear that, similar to analysis in Supplementary Note 3, we can draw the conclusion that as species equilibrium abundance increases, the stability of the system first increases, then decreases before finally losing stability entirely (Supplemental Fig. S12). Specifically, when $X^* \in [0, 1/(\tau s \mathrm{e}))$, stability increases as species abundance increases. When $X^* \in [1/(\tau s \mathrm{e}), \pi/(2\tau s))$, stability decreases as species abundance increases. When $X^* > \pi/(2\tau s)$, the system is unstable.

5.2.2 Multiple species community

As for multiple species communities, our results show that the non-monotonic relationship between species equilibrium abundance and stability also holds in multiple species community. We also explore how abundance affects the stability of communities with different interaction types (Supplemental Fig. S14). We find that when abundance is relatively low, exploitative communities possess the best stability performance. This is because the stability is determined by the rightmost eigenvalue in this stage (Supplemental Fig. S15). When abundance is relatively high, random communities are more stable than other communities since the distorted eigenvalue distributions of other types of communities make them more likely to reach the low stability region (Supplemental Fig. S15).

6 Supplementary Note 6: Understanding the biological meaning of time delay intensity

In former sections, we study the influence of an absolute time delay on ecosystem stability, which obscures its biological meaning. That is, it is not necessarily clear what a delay of $\tau = 1$ means in a real empirical ecosystem. To intuitively understand the biological meaning of time delay intensity, here we use the average doubling time of a random generalised Lotka-Volterra (gLV) system to normalise our previously arbitrary delay, τ.

6.1 Exponential growth and doubling time

When cultured in isolation with adequate resources, species experience exponential growth in abundance, which can be depicted by following equation [37]

$$\frac{\mathrm{d}X(t)}{\mathrm{d}t} = rX(t),$$

where r is the intrinsic growth rate of the species. Doubling time T_d is the time it takes for the species to double in abundance, and it can be theoretically derived for a species experiencing exponential growth [37]

$$T_d = \frac{\ln 2}{r}.$$

In the field of experimental biology, demography, and oncology, doubling time is often used to obtain a species' intrinsic growth rate. Here we use the average doubling time of a random gLV community to standardise the absolute time delay.

6.2 Understanding the biological meaning of time delay through doubling time

The dynamics of a gLV ecosystem is captured by following equation

$$\frac{\mathrm{d}\boldsymbol{X}(t)}{\mathrm{d}t} = \mathrm{diag}\left(\boldsymbol{X}(t)\right)\left(\boldsymbol{r} + \boldsymbol{A}\boldsymbol{X}(t)\right),$$

where $\boldsymbol{r} = [r_1, \cdots, r_S]^T$ is an S-dimensional vector whose element r_i is the intrinsic growth rate of species i, and $\boldsymbol{A}$ is the interaction matrix, whose elements A_{ij} depicts the per capita effect of species j on species i. The interaction matrix of a random gLV system is constructed by the method stated in Methods section. We assume the system rests at a homogeneous equilibrium $\boldsymbol{X}^* = \boldsymbol{1}$, which means

that $r_i - 1 + \sum_{j=1,j\neq i}^{S} A_{ij} = 0$. This leads to the expression of the intrinsic growth rate of species i

$$r_i = 1 - \sum_{j=1,j\neq i}^{S} A_{ij}.$$

For sufficiently large random gLV community, $\mathbb{E}(\sum_{j=1,j\neq i}^{S} A_{ij}) = 0$. We can obtain the average intrinsic growth rate: $\overline{r} = 1$. The average doubling time of a random gLV community is then

$$\overline{T}_d = \frac{\ln 2}{\overline{r}} = \ln 2.$$

Numerical simulations shown in Supplemental Fig. S16 verify our theoretical estimation of the doubling time.

By using $\overline{T}_d$ to normalise time delay, we get the relationship between stability and relative time delay (Figs. 3, 4, and Supplemental Figs. S17, S18). A crucial finding through this normalisation is that the stabilising effect of time delay occurs within a realistic range of delays, not only for infinitesimally small delays.

7 Supplementary Note 7: Scaling up complexity

Our main analyses are based on two simplifying assumptions: each member of the community experiences the same level of time delay in its population, and species interactions can be captured by the simple, direct net effects of species upon one another (e.g., the A_{ij} terms in the generalised Lotka-Volterra model).

In fact, different species may experience different time delays, and interactions between taxa are often mediated by external factors such as metabolites or toxins that give rise to complex, non-linear forms of interactions. This leads us to wonder whether our main result (i.e., moderate time delays have stabilising effect) will still hold without these two assumptions. Moreover, the influence of time delays is studied through explicitly introducing the time-delayed term in the main analyses, which is mathematically convenient and clear, but obscure the origin of the time delays in terms of underlying biological mechanisms. We also wonder if our main result still establishes when such explicit time-delayed terms are replaced by a specific biological process that can induce time-delayed scenarios. Here we relax each of these assumptions in turn.

7.1 Systems with heterogeneous time delays

In this section, we will first analyse the influence of heterogeneous delays on ecosystem stability within a simple 2-species case, and then discuss the challenges of the corresponding stability analysis for large complex ecosystems.

7.1.1 Model description

Without loss of generality, we model an ecosystem composed of S interacting species with heterogeneous delays by the generalised Lotka-Volterra model

$$\frac{\mathrm{d}X_i(t)}{\mathrm{d}t} = X_i(t)\left(r_i + \sum_{j=1}^{S} A_{ij}X_j(t-\tau_{ij})\right), \tag{S6}$$

where $X_i(t)$ represents the abundance of species i at time t, r_i depicts the intrinsic growth rate of species i, A_{ij} depicts the per capita interaction strength of species j on species i, and τ_{ij} is the time delay in the interaction of species j on species i.

The dynamical behavior of system Eq. (S6) around a feasible coexistence equilibrium $\boldsymbol{X}^*$ can be captured by the linearised equation

$$\frac{\mathrm{d}\boldsymbol{x}(t)}{\mathrm{d}t} = \sum_{i=1}^{S}\sum_{j=1}^{S} \boldsymbol{M}_{i,j}\boldsymbol{x}(t-\tau_{ij}),$$

where

$$\boldsymbol{M}_{1,1} = \begin{bmatrix} M_{11} & 0 & \cdots & 0 \\ 0 & 0 & \cdots & 0 \\ \vdots & \vdots & \ddots & \vdots \\ 0 & 0 & \cdots & 0 \end{bmatrix}, \boldsymbol{M}_{1,2} = \begin{bmatrix} 0 & M_{12} & \cdots & 0 \\ 0 & 0 & \cdots & 0 \\ \vdots & \vdots & \ddots & \vdots \\ 0 & 0 & \cdots & 0 \end{bmatrix}, \cdots,$$

$$\boldsymbol{M}_{S,S} = \begin{bmatrix} 0 & 0 & \cdots & 0 \\ 0 & 0 & \cdots & 0 \\ \vdots & \vdots & \ddots & \vdots \\ 0 & 0 & \cdots & M_{SS} \end{bmatrix},$$

the sum of these submatrices $\sum_{i=1}^{S}\sum_{j=1}^{S}\boldsymbol{M}_{i,j}$ is the original community matrix $\boldsymbol{M}$, and $\boldsymbol{x}(t) = \boldsymbol{X}(t) - \boldsymbol{X}^*$ is the deviation from equilibrium. Following the standard stability analysis framework of time-delayed systems, the stability can be determined by solving the following characteristic equation

$$\det\left(z\boldsymbol{I} - \sum_{i=1}^{S}\sum_{j=1}^{S}\boldsymbol{M}_{i,j}\mathrm{e}^{-\tau_{ij}z}\right) = 0, \tag{S7}$$

Provided all characteristic roots of Eq. (S7) have negative real parts, the system is stable. Moreover, the maximum real part of the characteristic roots can be an indicator of stability level.

7.1.2 Stability analysis of 2-species community

For a 2-species gLV system with heterogeneous delays, the governing equation becomes

$$\begin{cases} \frac{\mathrm{d}X_1(t)}{\mathrm{d}t} = X_1(t)\left(r_1 - s_1X_1(t-\tau_{11}) + A_{12}X_2(t-\tau_{12})\right), \\ \frac{\mathrm{d}X_2(t)}{\mathrm{d}t} = X_2(t)\left(r_2 - s_2X_2(t-\tau_{11}) + A_{21}X_2(t-\tau_{21})\right). \end{cases}$$

We still assume the system rests at a homogeneous equilibrium $\boldsymbol{X}^* = \mathbf{1}$, and the characteristic equation is now

$$z^2 + zs_1\mathrm{e}^{-z\tau_{11}} + zs_2\mathrm{e}^{-z\tau_{22}} + s_1s_2\mathrm{e}^{-z(\tau_{11}+\tau_{22})} - A_{12}A_{21}\mathrm{e}^{-z(\tau_{12}+\tau_{21})} = 0. \tag{S8}$$

By solving Eq. (S8) numerically, we can analyse the stability of this 2-species system.

Since we are now focusing on the influence of the heterogeneity of time delays on stability, we sample τ_{ij} from a Gamma distribution (to ensure we can obtain positive time delays) with mean τ_μ and variance τ_σ^2. The standard deviation τ_σ can then be an indicator of the heterogeneity of time delays.

As shown in Supplemental Fig. S19, the increase of the heterogeneity does harm to the stabilising effect of time delay. When the heterogeneity of time delays is relatively low, the increase of average strength of time delay (i.e., τ_μ) can still first stabilise and then destabilise the system. However, for relatively high heterogeneity, the increase of average strength has a uniform destabilising effect.

7.1.3 Challenge in the stability analysis of large complex ecosystems

Technically, the discussion can be extended to large complex ecosystems with heterogeneous delays. However, solving the characteristic equation for large complex ecosystems with heterogeneous delays is an NP-hard problem and is thus extremely time-consuming. For the case with homogeneous delays, we tackle this problem by recognising the stability contour plot in the complex plane and the eigenvalue distribution of the community matrix. This method holds a prerequisite that the explicit expression of the characteristic equation with respect to the eigenvalues of the community matrix can be obtained. However, such form of characteristic equation is hard to obtain with the presence of heterogeneous time delays. Thus, our stability estimation framework developed in the manuscript is not applicable to cases with heterogeneous delays, and we have to face the NP-hard problem directly. Therefore, determining the stability of complex ecosystems with heterogeneous delays is still an open problem.

7.2 Toy model of bacteria competition without explicit time delays

In our main text, we study the influence of time delays on ecological stability by explicitly introducing a time-delayed term (i.e., $\boldsymbol{X}(t-\tau)$) into the governing equations. That is, we use a delay differential equations (DDE) as opposed to an ordinary differential equations (ODE) form of the governing equa-

tions to capture system dynamics. This classic approach is convenient when exploring the general effect of time delays, and is mathematically clear. However, such governing equations (e.g., generalised Lotka-Volterra model) have been criticised for their simplicity, and this approach leaves open the origin of the time delays in terms of underlying biological mechanisms.

To address this, here we provide a more mechanistic model of microbial community dynamics to both illustrate how a specific biological scenarios can give rise to time-delayed processes, and demonstrate that our results hold when interactions between microbes take more complex functional forms. We build on a published model of microbial community dynamics in which interactions are mediated by nutrient competition and the production of antibacterial toxins (e.g., bacteriocins). As we show below, while this model is not an explicit delayed differential equation, one can model the impacts of delays in how species respond to one another's densities via the processes of nutrient and toxin uptake. Moreover, and importantly, we show that this more mechanistic modeling framework recapitulates the key findings of our general model presented in the main text.

7.2.1 Bacteria competition model without explicit time delays

We construct our more mechanistic model of bacterial community dynamics by extending a previous model developed by Niehus *et al.* [50]. In this model the growth of each bacterial species within a community is determined by their growth on a shared nutrient, and the impact of bacteriocins that are present in the environment due to the production by other community members. Importantly for our discussion, these models allow one to modulate the relationship between solute concentrations and the impacts of solutes on cell growth.

Specifically, the typical model is based on a Monod function, which assumes a saturating relationship between the concentration of a solute and cell growth rate. We can use this function to capture the impacts of delays driven by the need of each solute to first be transported into the cell and metabolised before it has an impact upon cell growth. Specifically, we do this with the term, β_i, which sets the rate at which the Monod function saturates. When $\beta_i = 0$, then there are no delays in the effects of solutes and thereby a simple linear relationship between solute concentrations and their impacts. To capture the impacts of delays in the impacts of species on one another, we set $\beta_i > 0$, which implies that the cell can no longer instantaneously use solutes at any concentration due to delays in update and metabolism. As shown in Supplemental Fig. S20, the time for species A to

reach its maximal deviation from equilibrium when increasing the biomass of species B is extended as β increases, indicating the interaction between species A and species B is delayed. This enables us to concurrently gain a more mechanistic understanding of the meaning of delays within our system, and assess whether our results hold when species interactions are defined by more complex functional forms. Notably, this functional form is also analogous to the Holling type II equations describing exploitative dynamics with delays introduced by predator handling time [2]. The dynamical behavior of this model can be captured by following equations

$$\begin{cases} \frac{\mathrm{d}S_A(t)}{\mathrm{d}t} = S_A(t)\left(r_A\left(1-P_A\right)\frac{N(t)}{1+\beta_1 N(t)} - \frac{S_A(t)+\varepsilon_1\frac{T_B(t)}{1+\beta_2 T_B(t)}}{K_A}\right), \\ \frac{\mathrm{d}S_B(t)}{\mathrm{d}t} = S_B(t)\left(r_B\left(1-P_B\right)\frac{N(t)}{1+\beta_3 N(t)} - \frac{S_B(t)+\varepsilon_2\frac{T_A(t)}{1+\beta_4 T_A(t)}}{K_B}\right), \\ \frac{\mathrm{d}T_A(t)}{\mathrm{d}t} = P_A\frac{N(t)}{1+\beta_1 N(t)}S_A(t) - l_T T_A(t), \\ \frac{\mathrm{d}T_B(t)}{\mathrm{d}t} = P_B\frac{N(t)}{1+\beta_2 N(t)}S_B(t) - l_T T_B(t), \\ \frac{\mathrm{d}N(t)}{\mathrm{d}t} = r_N - \frac{N(t)}{1+\beta_1 N(t)}S_A(t) - \frac{N(t)}{1+\beta_2 N(t)}S_B(t), \end{cases}$$

where $S_A(t)$ and $S_B(t)$ represent the biomass of each species at time t. $T_A(t)$ and $T_B(t)$ represent the biomass of each species' toxin at time t. $N(t)$ represents the abundance of nutrients. $r_A(t)$ and $r_B(t)$ are the intrinsic growth rates of each species. $r_N(t)$ is the rate at which nutrients flow into the environment. $P_A(t)$ and $P_B(t)$ denote the investment into toxin production relative to biomass. $K_A(t)$ and $K_B(t)$ denote the carrying capacity of each species. ε_1 and ε_2 are the killing efficiencies of each toxin. l_T denotes the degradation rate of toxin. β_1 and β_3 represent the time required for a unit nutrient to be taken up and metabolised by the bacteria cell. β_2 and β_4 represent the time for a unit toxin to be absorbed and take effect on cellular growth.

To simplify our analysis, we assume each species has the same carrying capacity (i.e., $K_A = K_B = K$), the handling time for each biological step is the same (i.e., $\beta_1 = \beta_2 = \beta_3 = \beta_4 = \beta_5 = \beta_6 = \beta$), and the killing efficiency of each toxin is the same (i.e., $\varepsilon_1 = \varepsilon_2$).

7.2.2 Evaluating the influence of time delays on stability

While our model no longer contains an explicit delay in the effect of species upon one another, it is clear that such delays will arise naturally depending upon the rate at which both nutrients and bacteriocins are absorbed into the bacterial cells. Therefore, next we assess the impact of changing these rates (i.e., β) upon community stability following the canonical stability analysis framework.

As shown in Supplemental Fig. S21, we find that the relation between the time for nutrients and bacteriocins to be absorbed/metabolised and stability is also non-monotonic, just as we revealed in the model with explicit delays: as the time necessary for nutrients/toxins to be absorbed increases, stability first increases, and then decreases. This result qualitatively confirms the results of our main analysis.

This non-monotonic relation can be understood through the influence of β on the elements of the community matrix $\boldsymbol{M}$. Just as we stated before, here we regard toxin and nutrients as general species. Therefore, off-diagonal elements depict interspecies interaction strengths, while diagonal elements represent self-regulation strengths. As toxin/nutrient absorption time increases, all interspecies interaction strengths and the self-regulation strength of the nutrients decrease. The decrease in interspecies interaction strength promotes system stability, while the decrease in self-regulation strength destabilises the system. When absorption time β is relatively low, although the self-regulation strength of the nutrients decreases, it is still higher than the self-regulation strength of other components (e.g., higher than the self-regulation of toxin). According to previous work on the influence of self-regulation [13], the influence of nutrients self-regulation strength is not obvious. Thus, the influence of interspecies interactions prevails, which leads to a stabilising effect. When absorption time β is relatively high, nutrients now have the lowest self-regulation strength and is thus the main driver of the stability of the toy model. This leads to a destabilising effect.

Supplementary References

[43] Nguyen, H. H. & O'Rourke, S. The elliptic law. *Int. Math. Res.* **2015**, 7620–7689 (2015).

[44] O'Rourke, S. & Renfrew, D. Low rank perturbations of large elliptic random matrices. *Electron. J. Probab.* **19**, 1–65 (2014).

[45] Tao, T., Vu, V. & Krishnapur, M. Random matrices: Universality of esds and the circular law. *Ann. Probab.* **38**, 2023–2065 (2010).

[46] Corless, R. M., Gonnet, G. H., Hare, D. E., Jeffrey, D. J. & Knuth, D. E. On the lambert W function. *Adv. Comput. Math.* **5**, 329–359 (1996).

[47] Hwang, C. & Cheng, Y.-C. A note on the use of the lambert W function in the stability analysis of time-delay systems. *Automatica* **41**, 1979–1985 (2005).

[48] Shinozaki, H. & Mori, T. Robust stability analysis of linear time-delay systems by lambert W function: Some extreme point results. *Automatica* **42**, 1791–1799 (2006).

[49] Jarlebring, E. & Damm, T. The lambert W function and the spectrum of some multidimensional time-delay systems. *Automatica* **43**, 2124–2128 (2007).

[50] Niehus, R., Oliveira, N. M., Li, A., Fletcher, A. G. & Foster, K. R. The evolution of strategy in bacterial warfare via the regulation of bacteriocins and antibiotics. *Elife* **10**, e69756 (2021).

Supplementary Figures

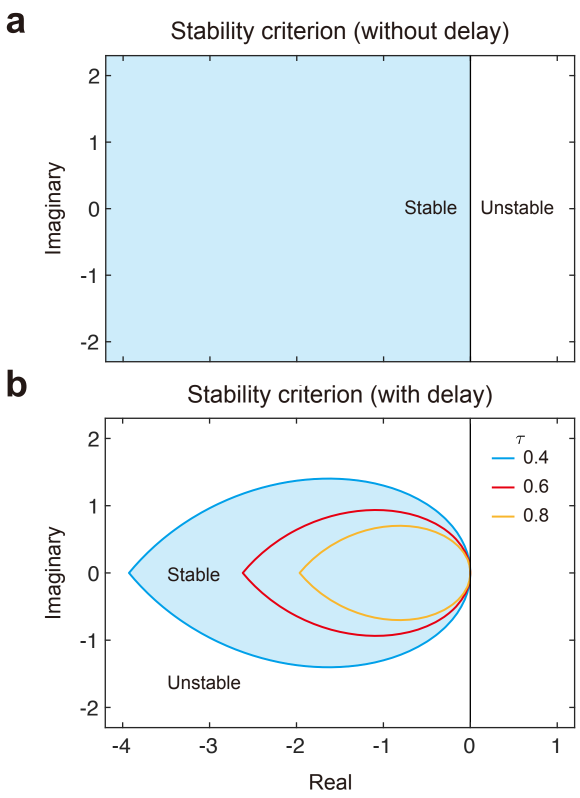

Supplemental Figure S1: a, Stability criterion for delay-free ecosystems. For delay-free ecosystems, all eigenvalues (λ) of the community matrix ($\boldsymbol{M}$ in Eq. (2)) should have negative real parts (i.e., $\mathrm{Re}(\lambda) < 0$) to ensure stability. The blue region in panel (a) represents the stability region. **b,** Stability criterion for time-delayed ecosystems, where all eigenvalues of the community matrix should distribute in the teardrop-shaped region to ensure stability. The blue region is the stability region for time delay $\tau = 0.4$. Black (vertical), blue, red and yellow lines indicate the boundaries for the stability region when $\tau = 0$, $\tau = 0.4$, $\tau = 0.6$, and $\tau = 0.8$, respectively.

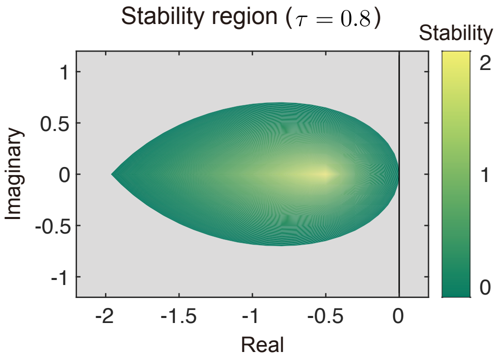

Supplemental Figure S2: Stability contour plot for ecosystems with time delay $\tau = 0.8$. Colours represent the value of $-\max(\mathrm{Re}(z))$, namely the stability level (see Methods and Supplementary Note 2). Colours closer to yellow represent larger $-\max(\mathrm{Re}(z))$, thus higher stability level. Colours closer to green indicate smaller $-\max(\mathrm{Re}(z))$, thus lower stability level. Grey colour represents positive $\max(\mathrm{Re}(z))$, thus instability.

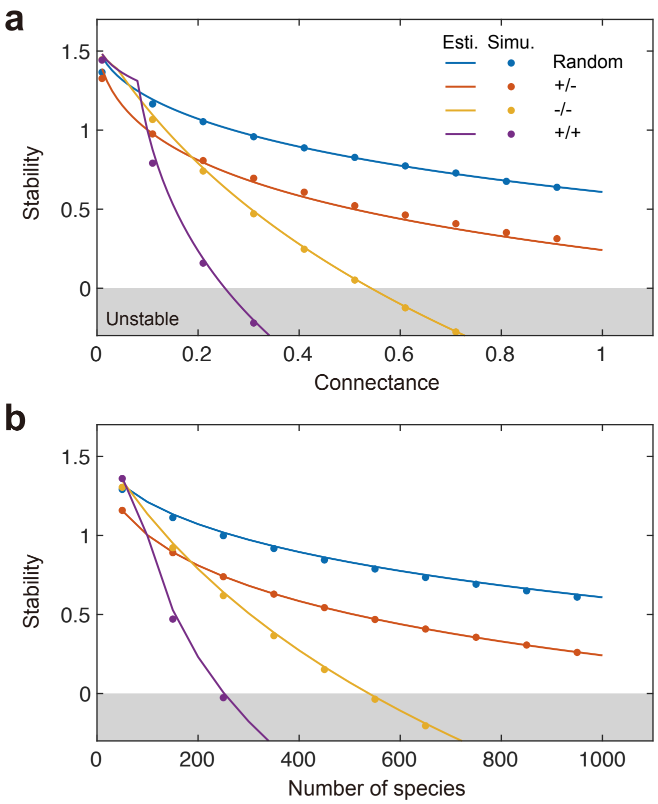

Supplemental Figure S3: Estimating the stability of communities with different levels of connectance (panel **a**), or numbers of species (panel **b**). Solid lines are estimations (Esti.) obtained from our framework (see Supplementary Note 2), and dots are obtained from numerical simulations (Simu.). Each dot is an average of 50 communities with the same set of community parameters. In (a), $S = 100$. In (b), $C = 0.1$. Other community parameters are the same as those in Fig. 3a.

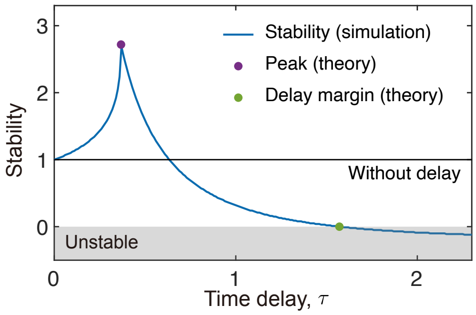

Supplemental Figure S4: Non-monotonic relationship between the intensity of time delay and community stability of a single species community. The blue line is the relationship between time delay and stability obtained from numerical calculations. The black horizontal line marks the level of stability without delay. The purple dot represents the peak predicted by our theory, and the green dot is the delay margin (i.e., maximum allowable time delay) predicted by our theory (see Supplementary Note 3). Community parameters are the same as those in Fig. 3b.

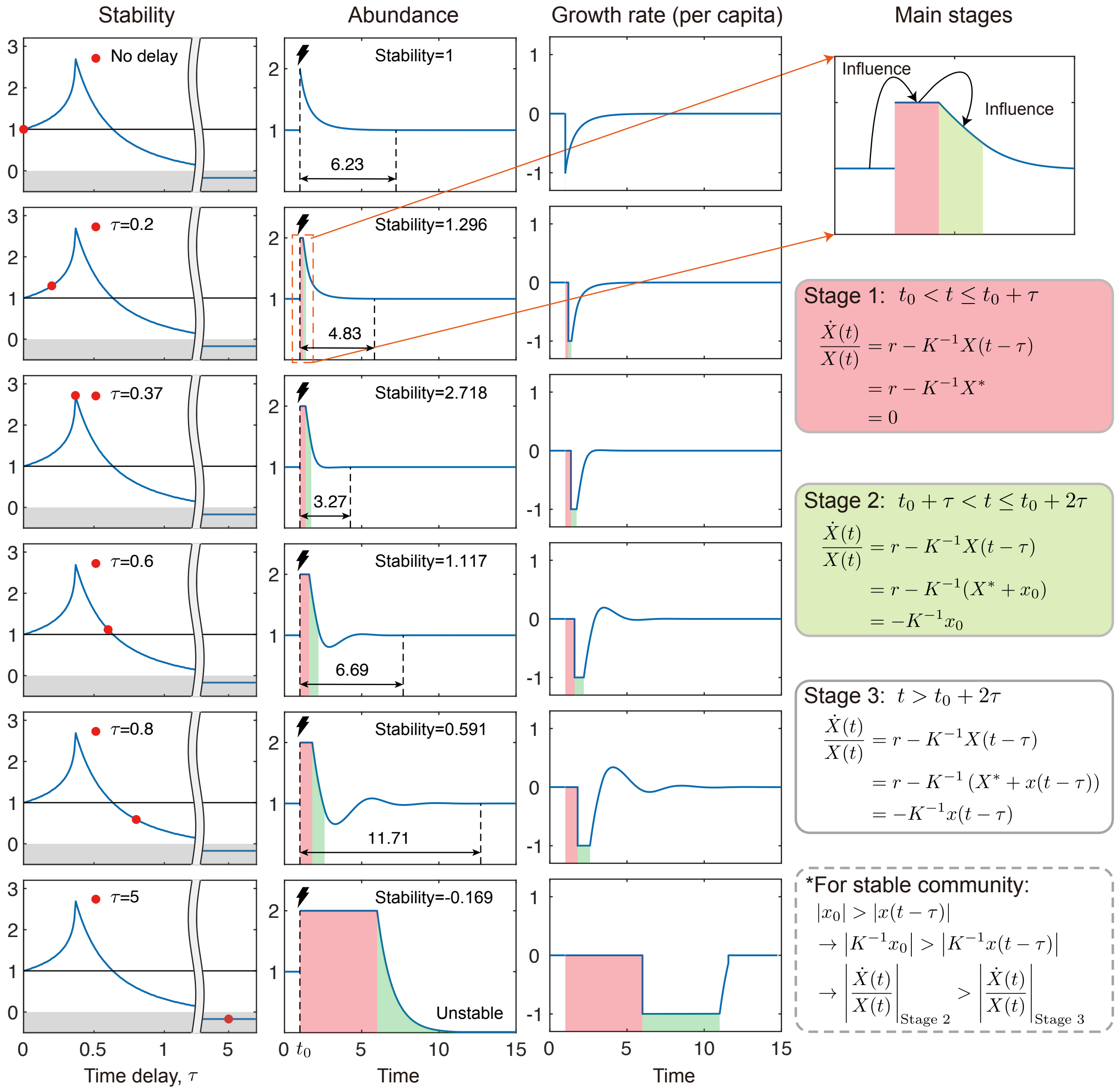

Supplemental Figure S5: Responses of a single species community with different time delays (τ) to external perturbations. Due to the delayed effect, the response process after perturbations can be divided into three stages according to per capita growth rate (detailed information is shown in figure). We here use logistic model with parameters: $r = 1$, $K = 1$, and equilibrium abundance is 1. See Fig. 3 for more information.

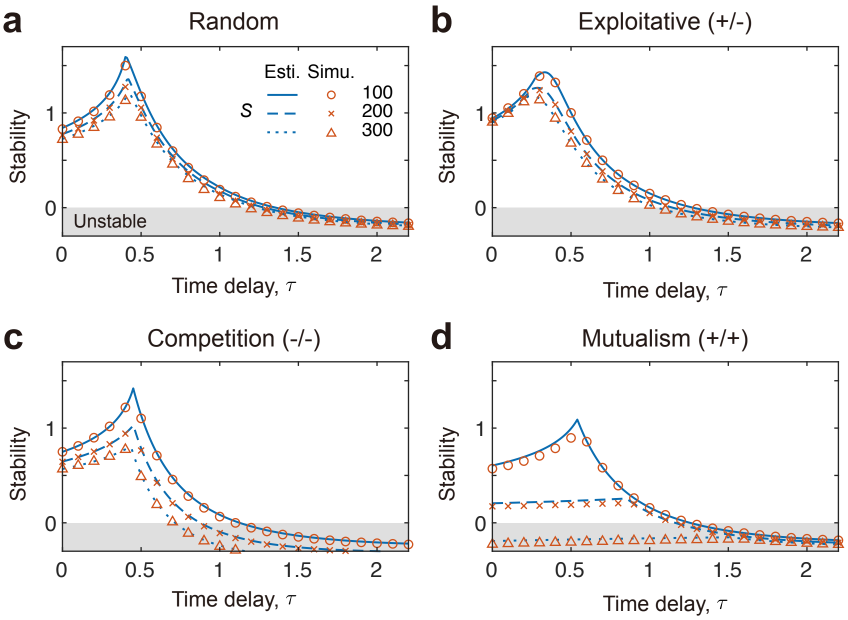

Supplemental Figure S6: Non-monotonic relationship between the intensity of time delay and the level of stability for different types of communities with different sizes (S). Blue lines are results from theoretical estimations (Esti.). Different markers are results from numerical simulations (Simu.), and each marker is an average of 50 simulations with the same set of community parameters. Other parameters are the same as those in Fig. 3a.

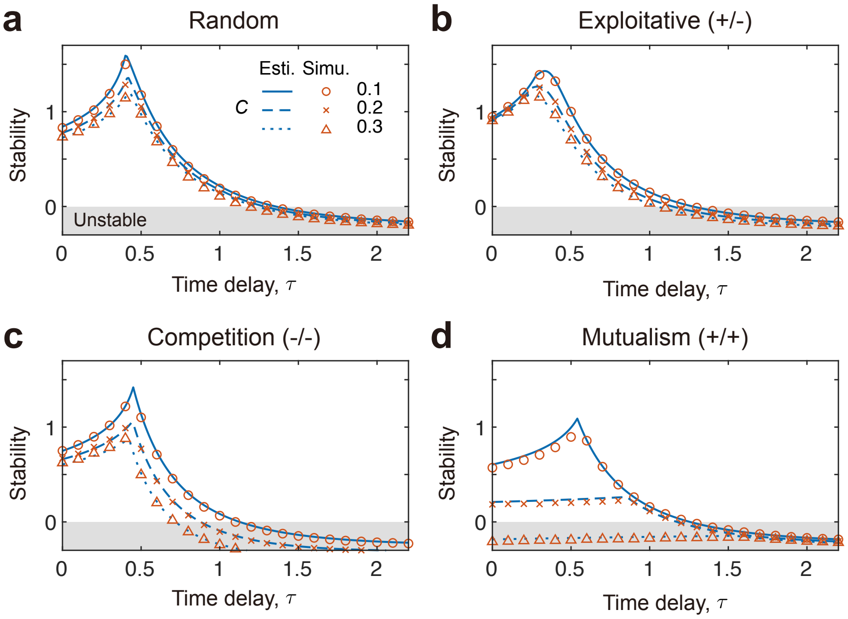

Supplemental Figure S7: Non-monotonic relationship between the intensity of time delay and the level of stability for different types of communities with different connectances (C). Blue lines are results from theoretical estimations (Esti.). Different markers are results from numerical simulations (Simu.), and each marker is an average of 50 simulations with the same set of community parameters. Other parameters are the same as those in Fig. 3a.

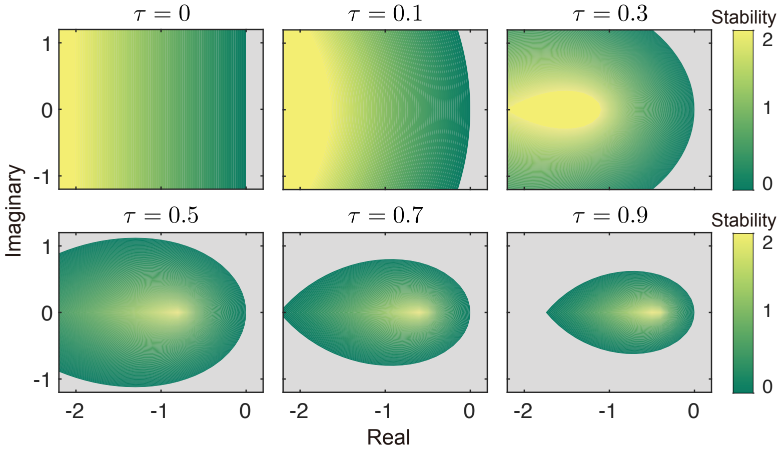

Supplemental Figure S8: Stability contour plot for different time delays (τ, see Supplementary Note 2). Colours represent the value of $-\max(\mathrm{Re}(z))$, and thus the degree of stability. Colours closer to yellow represents larger $-\max(\mathrm{Re}(z))$, thus higher stability level. Colours closer to green indicate smaller $-\max(\mathrm{Re}(z))$, thus lower stability level. Grey colour represents positive $\max(\mathrm{Re}(z))$, thus instability.

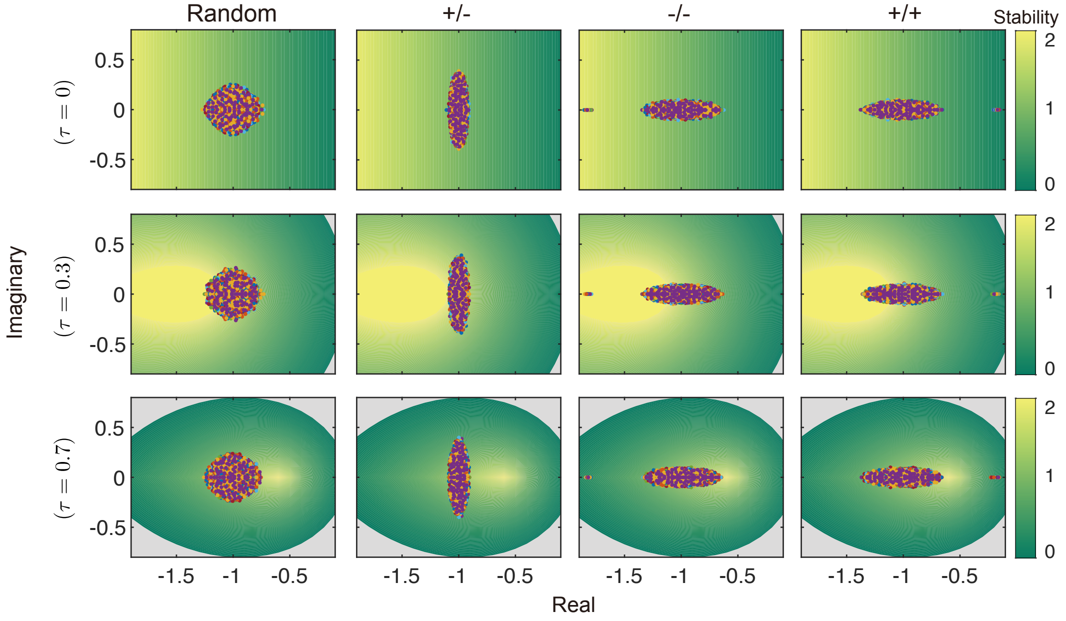

Supplemental Figure S9: With the presence of time delays, stability is no longer determined by the rightmost eigenvalue (see Supplementary Note 4). Community parameters are: $S = 100$, $C = 0.2$. Other settings are the same as those in Fig. 3a.

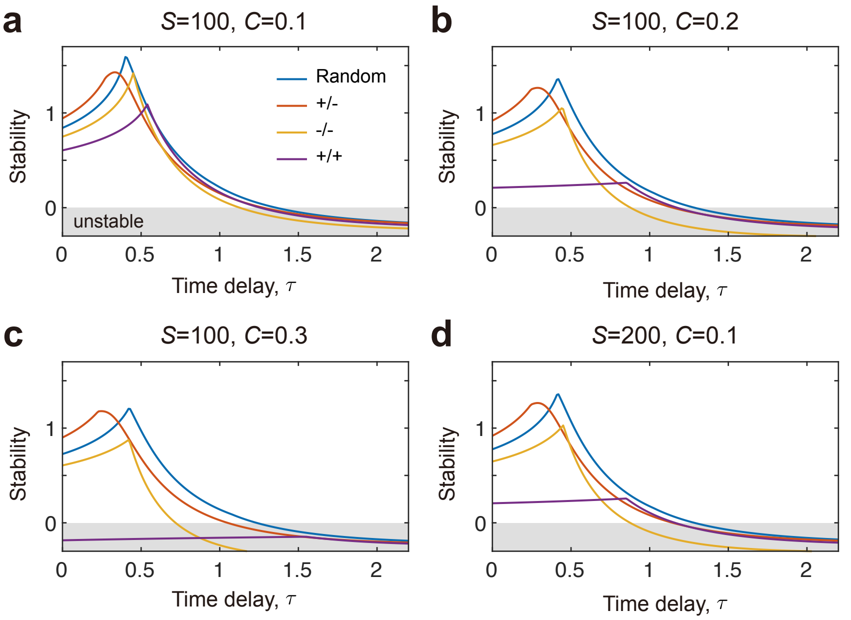

Supplemental Figure S10: When time delay is large, random communities (blue line in each panel) outperform other types of communities in terms of stability. Other community parameters are the same as those in Fig. 3a.

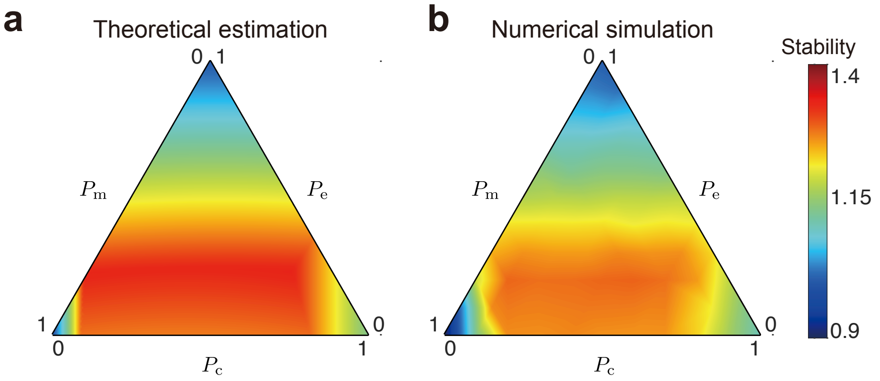

Supplemental Figure S11: A diversity of interaction types leads to a more stable community. We explore the stability of communities with mixed interactions of exploitation (P_{e}), competition (P_{c}), and mutualism (P_{m}). Panel **a** shows the result of theoretical estimation. Panel **b** shows the result from numerical simulations, and each datapoint is an average of 30 communities generated by the same set of community parameters. Note that in (a), the increasing step for the proportion of each interaction type is 0.01, while this increasing step in panel (b) is set to 0.1 to save time. Community parameters are the same as those in Fig. 3a, and we set time delay $\tau = 0.5$.

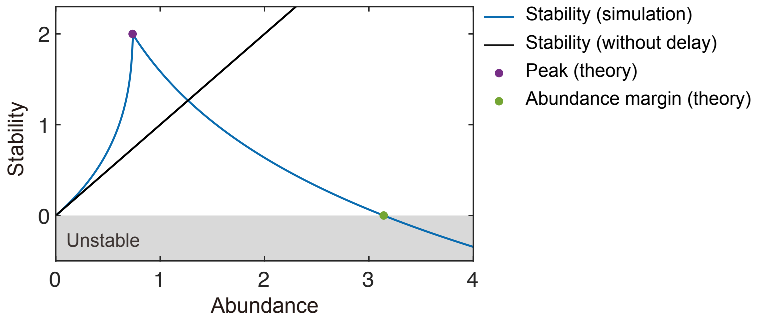

Supplemental Figure S12: Non-monotonic relationship between species equilibrium abundance and community stability of a single species community. The blue line is the relationship between stability and abundance obtained from numerical calculations with the presence of delay. The black line is this relation without delay. The purple dot is the peak predicted by our theory, and the green dot is the abundance margin (i.e., maximum allowable abundance) predicted by our theory (see Supplementary Note 5). Here we set $s = 1$, and time delay $\tau = 0.5$.

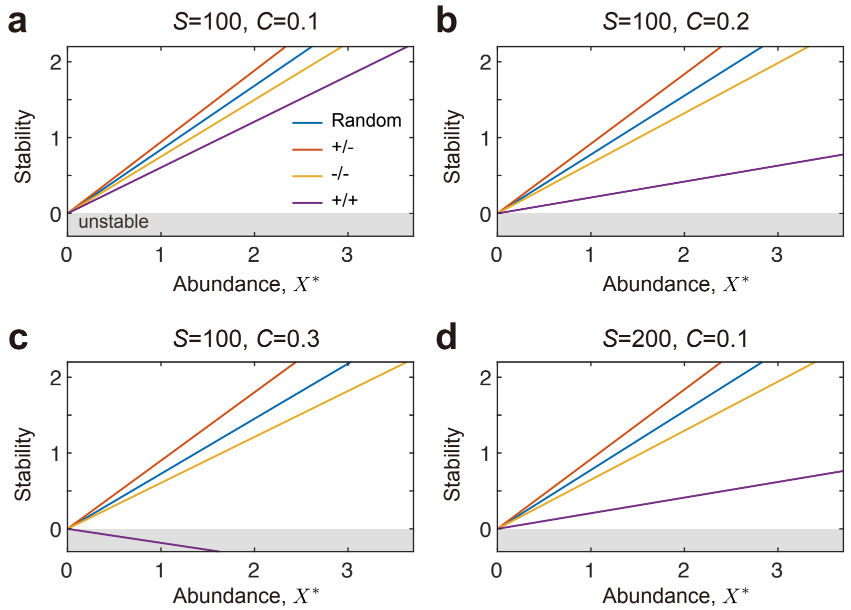

Supplemental Figure S13: The relationship between stability and species equilibrium abundance for delay-free multiple species communities. Note that all species share the same equilibrium abundance X^*. Other community parameters are the same as those in Fig. 3a.

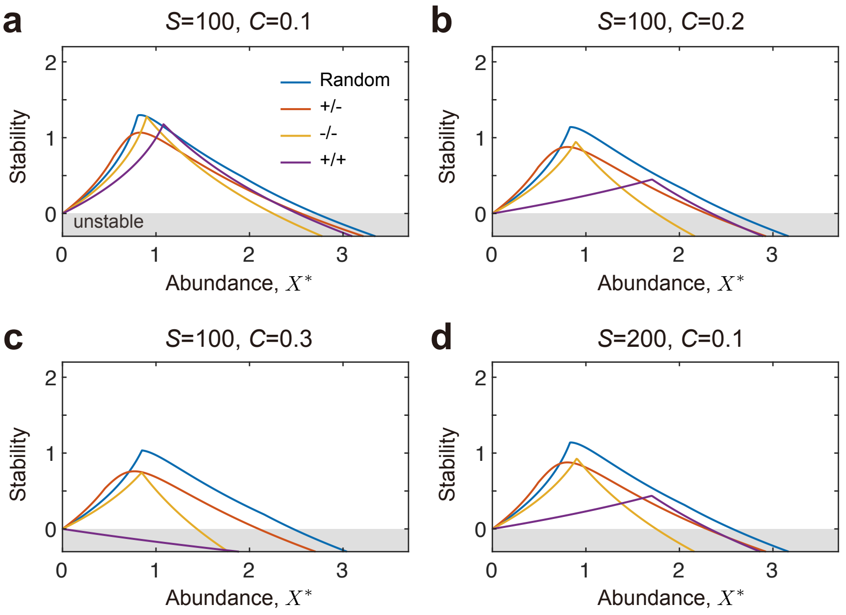

Supplemental Figure S14: For time-delayed communities, the relationship between stability and species abundance is non-monotonic: stability first increases and then decreases as abundance increases. Note that all species share the same equilibrium abundance X^*. Other community parameters are the same as those in Fig. 3a, and we set time delay $\tau = 0.5$.

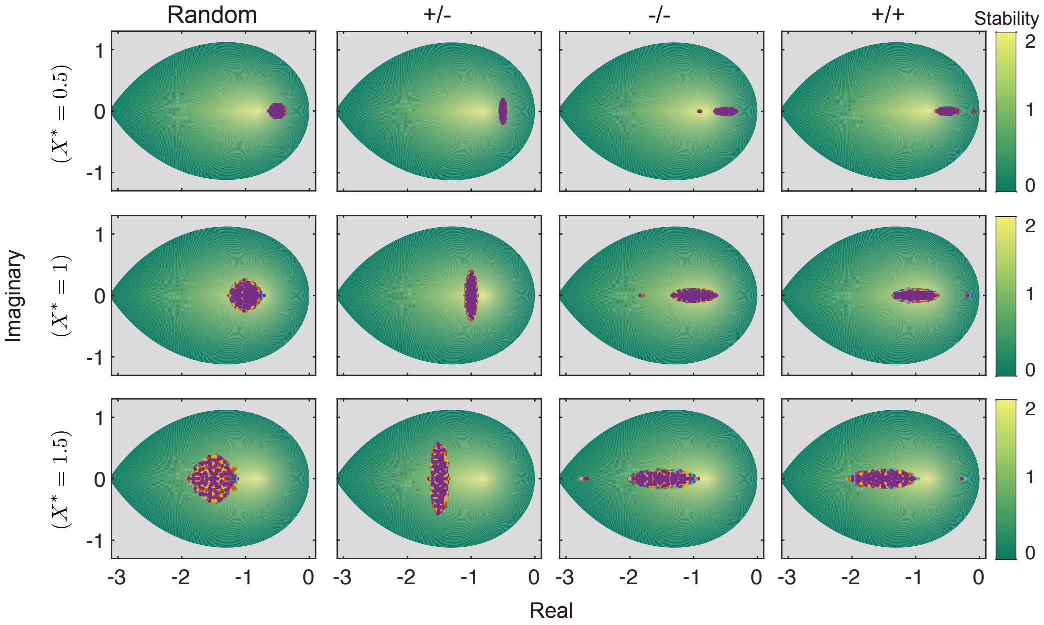

Supplemental Figure S15: Eigenvalue distributions of communities with different equilibrium abundances with the stability contour plot. Note that all species share the same equilibrium abundance X^*. Community parameters are the same as those in Fig. S9.

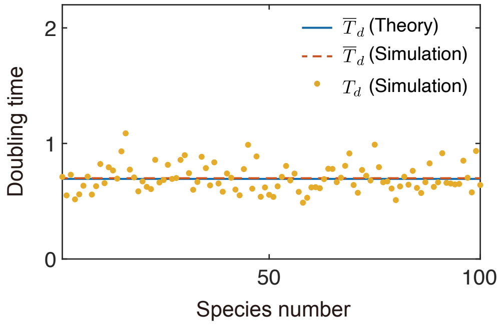

Supplemental Figure S16: Estimation of the average doubling time for random gLV ecosystems. Blue line is the average doubling time predicted by our theory, and red line is the average doubling time obtained from a community with 100 species. Yellow dots are the doubling time of each species in this community. Community parameters are the same as those in Fig. 3a.

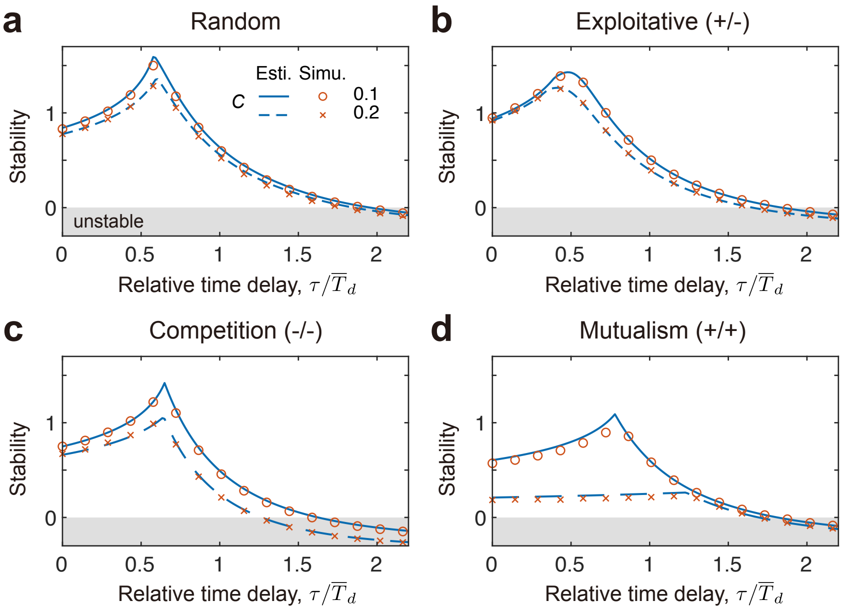

Supplemental Figure S17: The relationship between stability and relative time delay for different types of communities with different connectance C. Blue lines are results from theoretical estimations (Esti.). Different markers are results from numerical simulations and each one is an average of 50 communities with the same set of community parameter. Numerical simulations (Simu.) in this figure are based on gLV model with the same parameters as shown in Fig. S7.

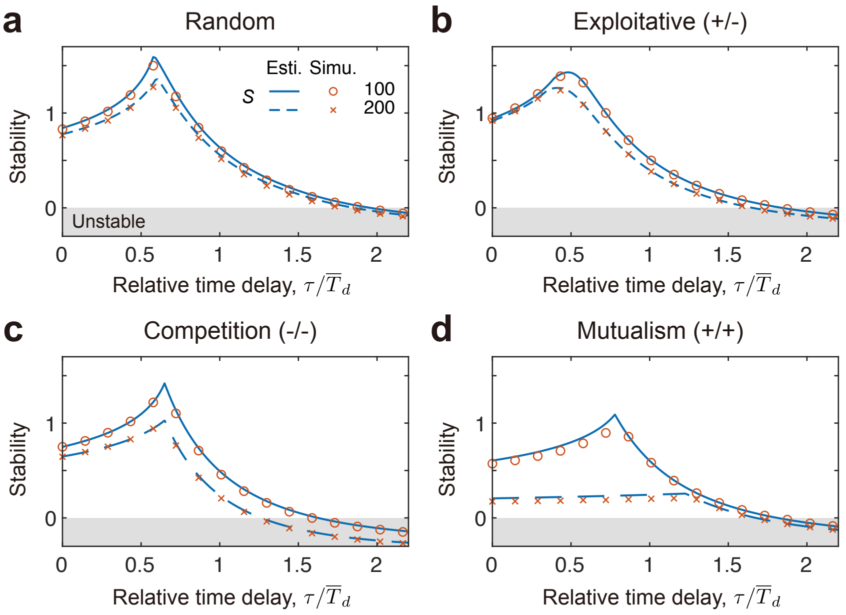

Supplemental Figure S18: The relationship between stability and relative time delay for different types of communities with different community sizes (S). Blue lines are results from theoretical estimations (Esti.). Different markers are results from numerical simulations and each one is an average of 50 communities with the same set of community parameter. Numerical simulations (Simu.) in this figure are based on gLV model with the same parameters as shown in Fig. S6.

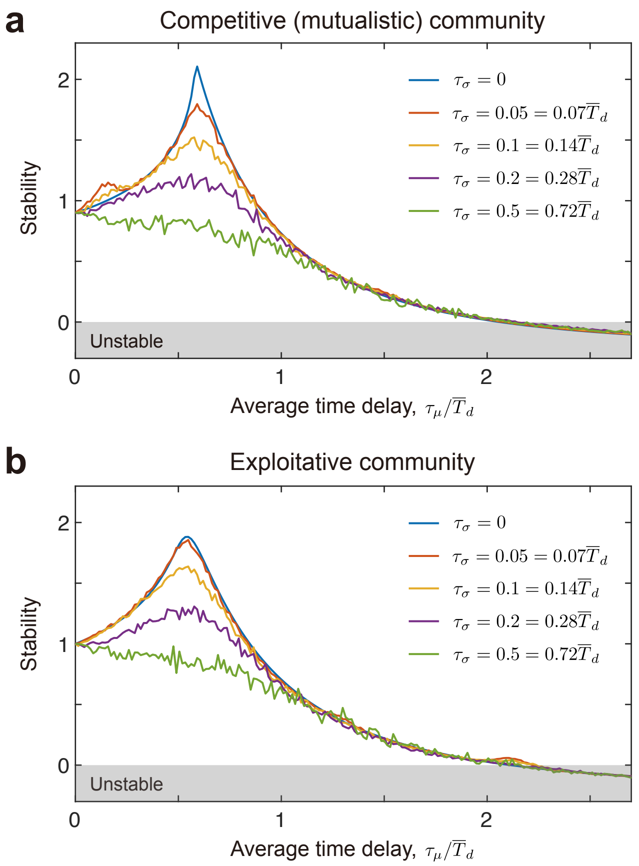

Supplemental Figure S19: Heterogeneity of time delays and stability of 2-species communities. The relationship between average time delay (i.e., τ_μ) and stability of 2-species competitive, mutualistic (panel **a**) and exploitative communities (panel **b**) under different heterogeneities (measured by τ_σ, corresponding to the standard deviation). Here we normalise average time delay by dividing average doubling time of the community. For community with heterogeneous delays, each datapoint is an average of 100 communities with the same time delay distribution parameters. Communities are modeled by the gLV model, with parameters $s_1 = s_2 = 1,\ |A_{12}| = |A_{21}| = 0.1$, and $X_1^* = X_2^* = 1$. Heterogeneous time delays are sampled from a Gamma distribution with mean τ_μ and variance τ_σ^2.

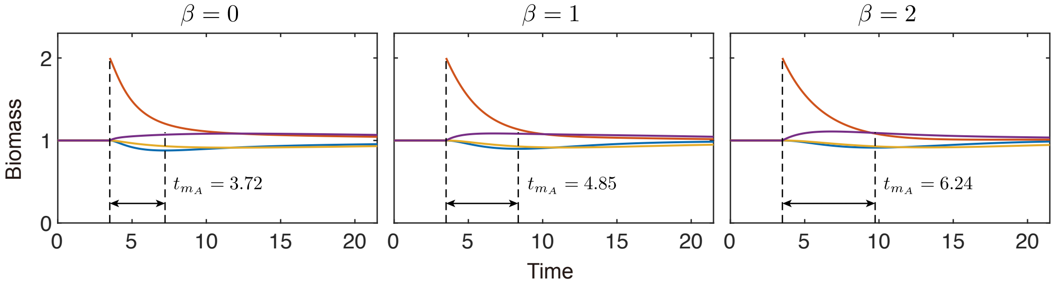

Supplemental Figure S20: Interspecies interaction is delayed due to the introduction of absorption time β (see Supplementary Note 7). The time for species A to reach maximum deviation from equilibrium (i.e., t_{m_A}) is extended as β increases, indicating the interaction between species A and species B is delayed. Community parameters are: $K = 5,\ \varepsilon = 0.1,\ l_T = 0.1$.

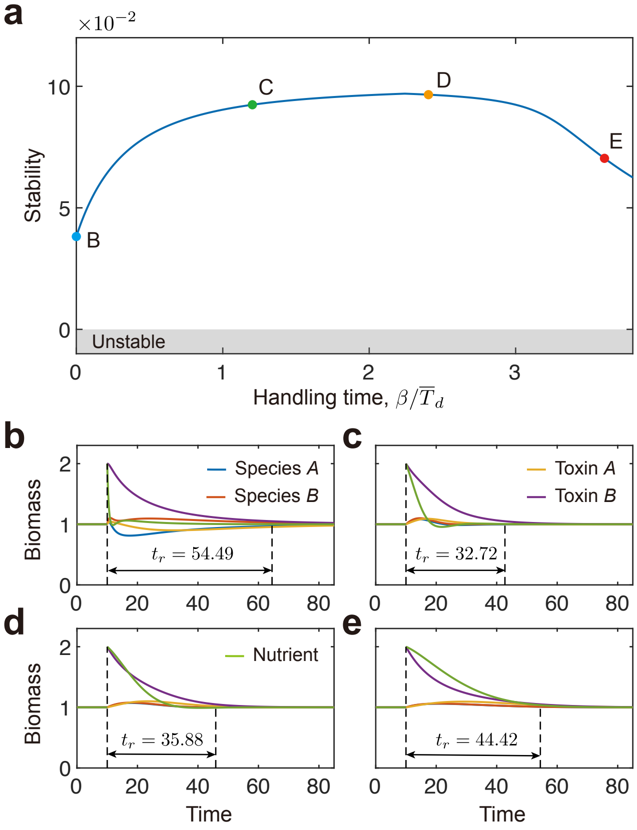

Supplemental Figure S21: Non-monotonic relationship between absorption time β and stability (see Supplementary Note 7). **a**, The relationship between absorption time β and stability. Here the time delay is normalised by the average doubling time of the community without absorption time. For different levels of handling time (dots b to e), we plot the response of the community after perturbation (**b** to **e**), and show the corresponding recovery time t_r in panels (b) to (e) separately. Community parameters are: $K = 5$, $\varepsilon = 0.1$, $l_T = 0.1$. In numerical simulations, we deem the community recoveries to the equilibrium if the deviation of each species' abundance from their equilibrium is less than 5%.

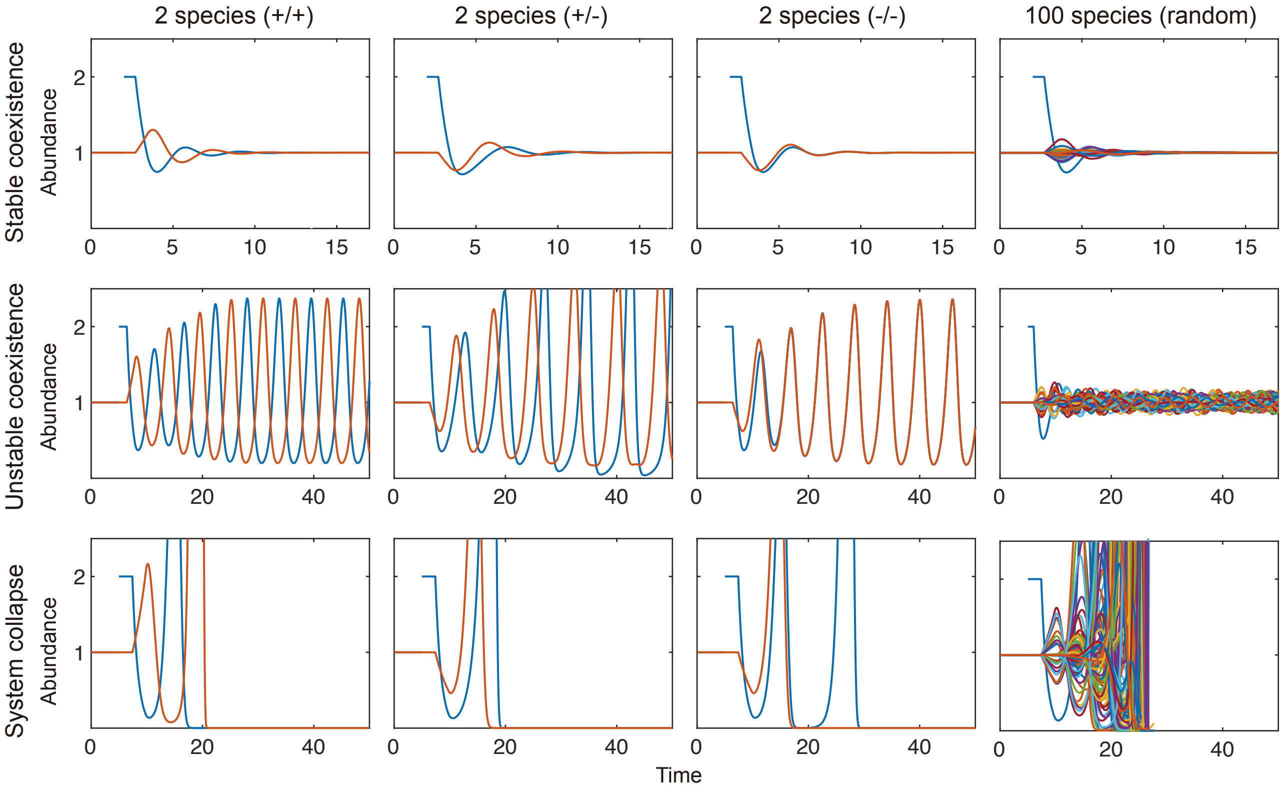

Supplemental Figure S22: Responses of different time-delayed communities to perturbation. As delay gradually increases, communities experience 3 stages: stable coexistence (top row), unstable coexistence (middle row) and system collapse stage (bottom row). In the stable coexistence stage, the feasible coexistence equilibrium maintains linear stability, and system can recover from perturbations. In the unstable coexistence stage, the feasible coexistence equilibrium loses linear stability, and the system enters an oscillatory regime. However, species can still coexist in this stage. In the system collapse stage, species cannot coexist, and some species go extinct. Simulations are based on the gLV model, and community parameters are as follows. For 2 species mutualistic communities, we set $A_{12} = A_{21} = 0.3,\ r_1 = r_2 = 0.7,\ s_1 = s_2 = 1$. For 2 species exploitative communities, we set $A_{12} = 0.3,\ A_{21} = -0.3,\ r_1 = 0.7,\ r_2 = 1.3,\ s_1 = s_2 = 1$. For 2 species competitive communities, we set $A_{12} = A_{21} = -0.3,\ r_1 = r_2 = 1.3,\ s_1 = s_2 = 1$. For random community with 100 species, $\boldsymbol{A}$ is constructed as is presented in the Methods section with parameters $S = 100,\ C = 0.2,\ s = 1,\ \sigma = 0.1$. For 2 species communities, time delays in each stage are 0.7 (stable coexistence stage), 1.4 (unstable coexistence stage) and 2.4 (system collapse stage), respectively. For random community with 100 species, time delays in each are 0.7 (stable coexistence stage), 1.04 (unstable coexistence stage) and 2.4 (system collapse stage), respectively.
